# Production of 3′,3′-cGAMP by a *Bdellovibrio bacteriovorus* promiscuous GGDEF enzyme, Bd0367, regulates exit from prey by gliding motility

**DOI:** 10.1371/journal.pgen.1010164

**Published:** 2022-05-27

**Authors:** Rebecca C. Lowry, Zachary F. Hallberg, Rob Till, Tyler J. Simons, Ruth Nottingham, Fiona Want, R. Elizabeth Sockett, Ming C. Hammond, Carey Lambert

**Affiliations:** 1 School of Life Sciences, University of Nottingham, Medical School, Nottingham, United Kingdom; 2 University of California, Berkeley, Department of Chemistry, Berkeley, California, United States of America; 3 University of Utah, Department of Chemistry and Center for Cell & Genome Science, Salt Lake City, Utah, United States of America; Michigan State University, UNITED STATES

## Abstract

Bacterial second messengers are important for regulating diverse bacterial lifestyles. Cyclic di-GMP (c-di-GMP) is produced by diguanylate cyclase enzymes, named GGDEF proteins, which are widespread across bacteria. Recently, hybrid promiscuous (Hypr) GGDEF proteins have been described in some bacteria, which produce both c-di-GMP and a more recently identified bacterial second messenger, 3′,3′-cyclic-GMP-AMP (cGAMP). One of these proteins was found in the predatory *Bdellovibrio bacteriovorus*, Bd0367. The *bd0367* GGDEF gene deletion strain was found to enter prey cells, but was incapable of leaving exhausted prey remnants via gliding motility on a solid surface once predator cell division was complete. However, it was unclear which signal regulated this process. We show that cGAMP signalling is active within *B*. *bacteriovorus* and that, in addition to producing c-di-GMP and some c-di-AMP, Bd0367 is a primary producer of cGAMP *in vivo*. Site-directed mutagenesis of serine 214 to an aspartate rendered Bd0367 into primarily a c-di-GMP synthase. *B*. *bacteriovorus* strain *bd0367*S214D phenocopies the *bd0367* deletion strain by being unable to glide on a solid surface, leading to an inability of new progeny to exit from prey cells post-replication. Thus, this process is regulated by cGAMP. Deletion of *bd0367* was also found to be incompatible with wild-type flagellar biogenesis, as a result of an acquired mutation in flagellin chaperone gene homologue *fliS*, implicating c-di-GMP in regulation of swimming motility. Thus the single Bd0367 enzyme produces two secondary messengers by action of the same GGDEF domain, the first reported example of a synthase that regulates multiple second messengers *in vivo*. Unlike roles of these signalling molecules in other bacteria, these signal to two separate motility systems, gliding and flagellar, which are essential for completion of the bacterial predation cycle and prey exit by *B*. *bacteriovorus*.

## Author summary

Secondary messengers are important signalling molecules in bacteria and a recently discovered one, called cGAMP, has recently been shown to be made by some enzymes which had previously been known to produce another secondary messenger, c-di-GMP. One of these “hybrid promiscuous” enzymes (Bd0367) is found in *Bdellovibrio bacteriovorus*, a bacterium that preys upon other bacteria, burrowing inside them and consuming them from within. Previous gene deletion work had shown that Bd0367 was essential in signalling for *Bdellovibrio* to leave the remains of its prey cell by gliding motility after predation was complete and it was thought that this was due to c-di-GMP signalling. However, here, we show that this gliding motility is actually regulated by cGAMP signalling and that c-di-GMP signalling is involved in swimming motility. A single enzyme produces two different molecules, signalling to two discrete motility systems, both of which are required for successful completion of the bacterium’s predatory lifestyle in prey on solid surfaces or in liquids.

## Introduction

Three cyclic dinucleotide (CDN) second messengers have been studied in bacteria to date: the best characterised is cyclic di-GMP (c-di-GMP), which is produced by diguanylate cyclase enzymes (or GGDEF proteins, named after a conserved catalytic motif) and is involved in the control of a variety of cellular processes, such as the regulation of motile versus sessile bacterial lifestyles (biofilm formation) [1,2]. Another second messenger is cyclic di-AMP (c-di-AMP) which has been found to regulate a number of processes in Gram-positive bacteria, such as osmotic shock response, cell wall homeostasis and sporulation [3,4]. More recently, 3’,3’-cyclic-GMP-AMP (cGAMP) has also been discovered in bacteria. It was originally identified in *Vibrio cholerae* to have a role in virulence (motility and intestinal colonisation in mice) [5] and to be produced by the cGAMP synthase, DncV. Since its discovery, cGAMP has also been found to regulate processes in non-pathogenic bacteria, including the bacterial CBASS system where cGAMP synthesis promotes *E*. *coli* cell death upon bacteriophage foreign DNA injection to prevent phage replication [6,7].

It was also found that *Geobacter* spp, use a cGAMP-sensing riboswitch to control genes associated with exoelectrogenesis [8,9]. No homologues of the *V*. *cholerae* cGAMP synthase, DncV, are present in *Geobacter*. Instead, cGAMP is produced by hybrid promiscuous (Hypr) GGDEF proteins that also possess c-di-GMP and c-di-AMP synthase activity [10]. That study also found that these Hypr GGDEF proteins are present in a number of other bacteria, although they are relatively rare, making up only 0.17% of all GGDEF domains [11].

The well characterised *Caulobacter* GGDEF protein, PleD, contains an aspartate residue (D344) in its GGDEF domain involved in GTP interactions during c-di-GMP synthesis and this aspartate residue is conserved in strict c-di-GMP producers [12]. However, variation of this residue in the GGDEF domain of Hypr GGDEF proteins, to either a serine or threonine, allows this domain to have specificity for both ATP and GTP [10]. Specificity for GTP is restored in the mutated Hypr GGDEF protein from *G*. *sulfurreducens*, GacAS347D (GSU1658), confirming this to be the specificity residue required for cGAMP synthesis. When this residue was mutated to an aspartate (as is present in PleD), this protein was only able to produce c-di-GMP. Interestingly, a Hypr GGDEF protein exists in the predatory bacterium *Bdellovibrio bacteriovorus* HD100, Bd0367. *B*. *bacteriovorus*, when living host-dependently and in a primed state known as ‘attack-phase’, has the ability to seek out other Gram-negative prey cells, either with rapid swimming motility in liquid environments (with speeds up to 160 μm/s being observed) [13] or via gliding motility on solid surfaces [14]. They then attach to their prey via a type IVa pilus-extruding predatory pole, invade and replicate within prey cells which they round up and kill (forming a composite structure known as a bdelloplast). The host-dependent (HD) replication process ends with the replicated *B*. *bacteriovorus* progeny bursting from the nutrient-depleted bdelloplast, in attack phase, ready to invade other prey cells [15].

Cyclic-di-GMP signaling from several GGDEF proteins has roles in controlling both predatory and non-predatory lifecycles, with three GGDEF family proteins being expressed inside predatory cells of *B*. *bacteriovorus* [16] and so the possibility of cGAMP signalling also being present in *B*. *bacteriovorus* is particularly intriguing. *B*. *bacteriovorus* strains can also live prey/host-independently (HI), and although mutations in genes permitting host-independent replication are rare (arising 1 in 10^7^) [17,18], this lifestyle can be used under axenic laboratory conditions to rescue and analyse non-predatory *B*. *bacteriovorus* mutants. The ability of *B*. *bacteriovorus* to kill a wide variety of Gram-negative prey, including pathogens of humans, plants and animals, has led to research into its potential as a biohazard control agent and its possible use in the fight against antimicrobial resistance [19–23]. Therefore, gaining a full understanding of predatory mechanisms and their control is crucial for the potential use of this bacterium as a live therapeutic.

A number of c-di-GMP signalling proteins were previously reported to be present in *B*. *bacteriovorus*: 5 GGDEF (comprising DgcA-D and degenerate GGDEF CdgA), 1 EAL and 6 HD-GYP domain-containing phosphodiesterases, and 18 predicted PilZ domain proteins that potentially bind c-di-GMP [16,24]. Previous mutational studies of the genes encoding the *B*. *bacteriovorus* GGDEF proteins established that separate pathways control predatory vs axenic lifestyles and also gliding motility [16]. One GGDEF protein, DgcB, was involved in prey entry and the Δ*dgcB* deletion strain was non-invasive, could only be isolated host-independently, and was shown by Meek and coworkers to mechanistically respond to physical deformation via an FHA domain [25]. Conversely, DgcC was involved in the transition to the host-independent lifestyle with the Δ*dgcC* mutant being an obligate predator and unable to grow host-independently. The Hypr GGDEF protein, Bd0367 (DgcA), was found previously, by deletion mutagenesis, to have a role in gliding motility and escape from bdelloplasts by Hobley and coworkers [16]. The Δ*bd0367* deletion strain could only be isolated host-independently (axenically), and did not complete the predatory process, although prey invasion could occur and was observed by application of axenically grown *B*. *bacteriovorus* to prey. At the time, control of these processes was attributed to c-di-GMP signalling and, therefore, the diguanylate cyclase activity of this protein.

Here we show that the second messenger, cGAMP, is present within *B*. *bacteriovorus* and that the Hypr GGDEF protein, Bd0367, is primarily responsible for its production in this predatory bacterium (in addition to producing c-di-GMP and c-di-AMP). S214D site-directed mutagenesis, changing Bd0367 to a c-di-GMP synthase, revealed that this *B*. *bacteriovorus* strain phenocopies the Δ*bd0367* deletion strain, implicating cGAMP signalling in predatory lifecycle control by initiating progressive gliding motility required for progeny escape from the bdelloplast on a solid surface; events crucial to completing the host-dependent predatory lifecycle. The control of *B*. *bacteriovorus* predatory processes is more complex than previously imagined with interplay from both c-di-GMP and cGAMP signalling.

## Results

### *B*. *bacteriovorus* Bd0367 protein produces cGAMP while a S214D variant does not

**Fig 1:**
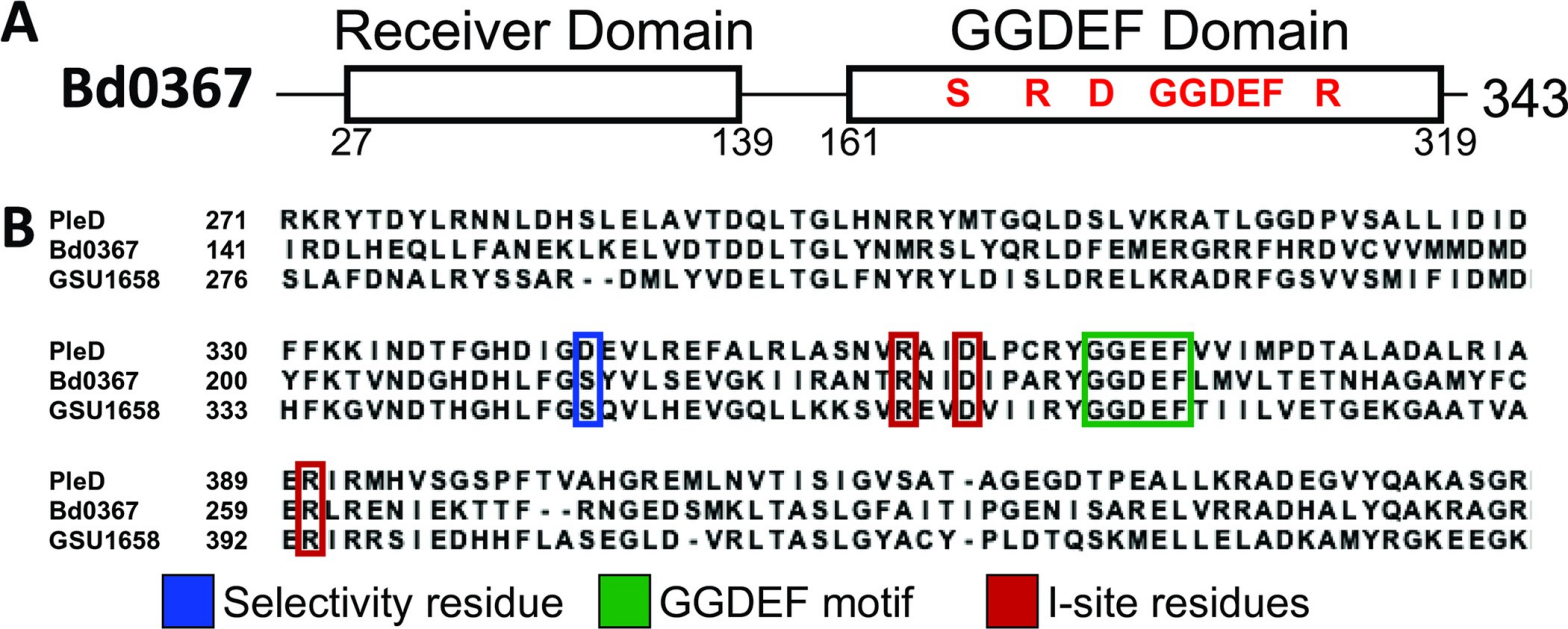
Bioinformatic analysis of Bd0367. **A-** Diagrammatic representation of the domains of *B*. *bacteriovorus* Bd0367. **B-** Alignment of the primary amino acid sequence of the GGDEF domains of *Caulobacter vibriodes* (CB15) PleD, *Bdellovibrio bacteriovorus* Bd0367, and *Geobacter sulfurreducens* GacA(GSU1658). The selectivity residue (blue) differs in the Hypr proteins (Bd0367 and GSU1658) where a serine residue is present, compared to the aspartate residue at position 344 in a canonical GGDEF enzyme such as PleD. Residues involved in the GGDEF domain (green) and I-site domains (red) are also highlighted.

**Fig 2:**
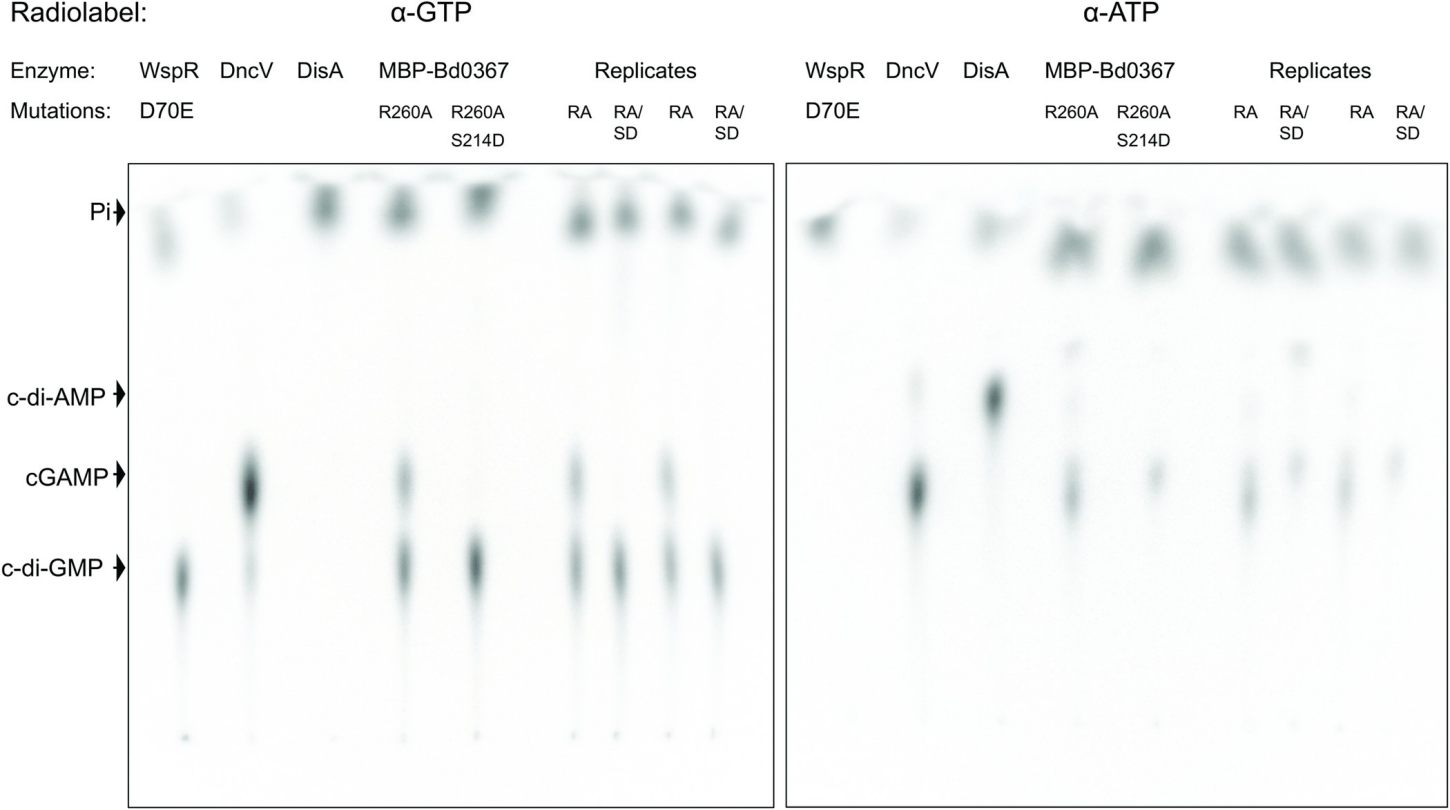
Bd0367 produces different cyclic dinucleotides. Cellulose TLC analysis of radiolabeled products from enzymatic reactions with 1:1 ATP-to-GTP substrates in excess and doped with trace amounts of α-^32^P-labeled GTP (left panel) or α-^32^P-labeled ATP (right panel). Before loading, reactions were quenched with alkaline phosphatase to digest unreacted nucleotides, resulting in production of inorganic phosphate (Pi). Residue R260 of Bd0367 is located in the putative I-site. Residue S214 is located at the selectivity position identified in previous studies and is necessary for cGAMP, but not c-di-GMP, production as mutants (S214D, SD) showed no bands at the expected position (cGAMP) whereas positive controls (DncV and MBP-Bd0367 R260A) did. WspR D70E is a positive control for c-di-GMP production and negative control for cGAMP production. DisA is a positive control for c-di-AMP production and negative control for cGAMP production.

Hallberg *et al*. [10] identified Bd0367 from *B*. *bacteriovorus* HD100 as a hybrid promiscuous (Hypr) GGDEF enzyme and demonstrated *in vitro* that Bd0367 functions as a cGAMP synthase as well as a diguanylate cyclase. Substrate specificity is predicted to be determined by a conserved serine residue in the GGDEF domain of Hypr proteins (S214 in Bd0367; **Fig 1**) in place of a key aspartate residue present in the majority of GGDEF proteins. Canonical GGDEF domains also have a conserved allosteric inhibitory site (I-site) that binds cyclic dinucleotides, and this is conserved in Bd0367 (including key residue R260; **Fig 1**). Hallberg *et al*. previously showed that wild-type GacA from *Geobacter sulfurreducens* co-purifies predominantly with c-di-GMP bound and has low activity when expressed in *E*. *coli*, whereas the R393A I-site mutant of GacA does not purify with bound dinucleotides and exhibits increased *in vitro* activity [10]. Similarly, MBP-tagged Bd0367 co-purifies with c-di-GMP and cGAMP, whereas the R260A I-site mutant of Bd0367 does not (**Figs A-C in S1 Text**). We therefore used this I-site mutant (R260A) to assess the *in vitro* activity of Bd0367 and introduced a further S214D mutation to demonstrate the residue’s role in substrate discrimination. Radiolabeled product analysis showed that Bd0367 (R260A) was capable of producing both c-di-GMP and cGAMP *in vitro*, whereas the S214D mutant has switched to producing almost exclusively c-di-GMP (**Fig 2**). Further product analysis using LC-MS was performed (**Fig B in S1 Text**), and showed that Bd0367 (R260A) produces primarily cGAMP, followed by c-di-GMP, with c-di-AMP also observed as a minor product. As expected, this enzyme produces 3’,3’-cGAMP and not any of the other linkage isomers (2’,3’ or 3’,2’) [26]. Similar analysis of the S214D mutant confirms that it produces almost exclusively c-di-GMP, and thus S214 is important for cGAMP production by Bd0367.

### Cyclic dinucleotide analysis of cell extracts reveals that cGAMP is produced in *B*. *bacteriovorus*, *in vivo*, with Bd0367 being the sole synthase

To investigate the role of cGAMP in *B*. *bacteriovorus*, site-directed mutagenesis of the *bd0367* gene was carried out whereby the serine residue (S214) was mutated to an aspartate residue, to investigate whether cGAMP is being produced in *B*. *bacteriovorus* by Bd0367 and to explore any phenotypic effects of this mutagenesis. The mutant gene, *bd0367*(S214D), was inserted into the *B*. *bacteriovorus bd0367* deletion strain (*Δbd0367*) at the wild type locus. Complementation strains were also generated for both Δ*bd0367* (by inserting the wild type gene into the site of deletion) and *bd0367*(S214D) strains (by replacing the site-directed mutant gene with the wild type gene inserted into the genome). The strains generated here were all cultured host-independently as they were based on the original Δ*bd0367* strain produced by Hobley *et al*. [16].

**Fig 3:**
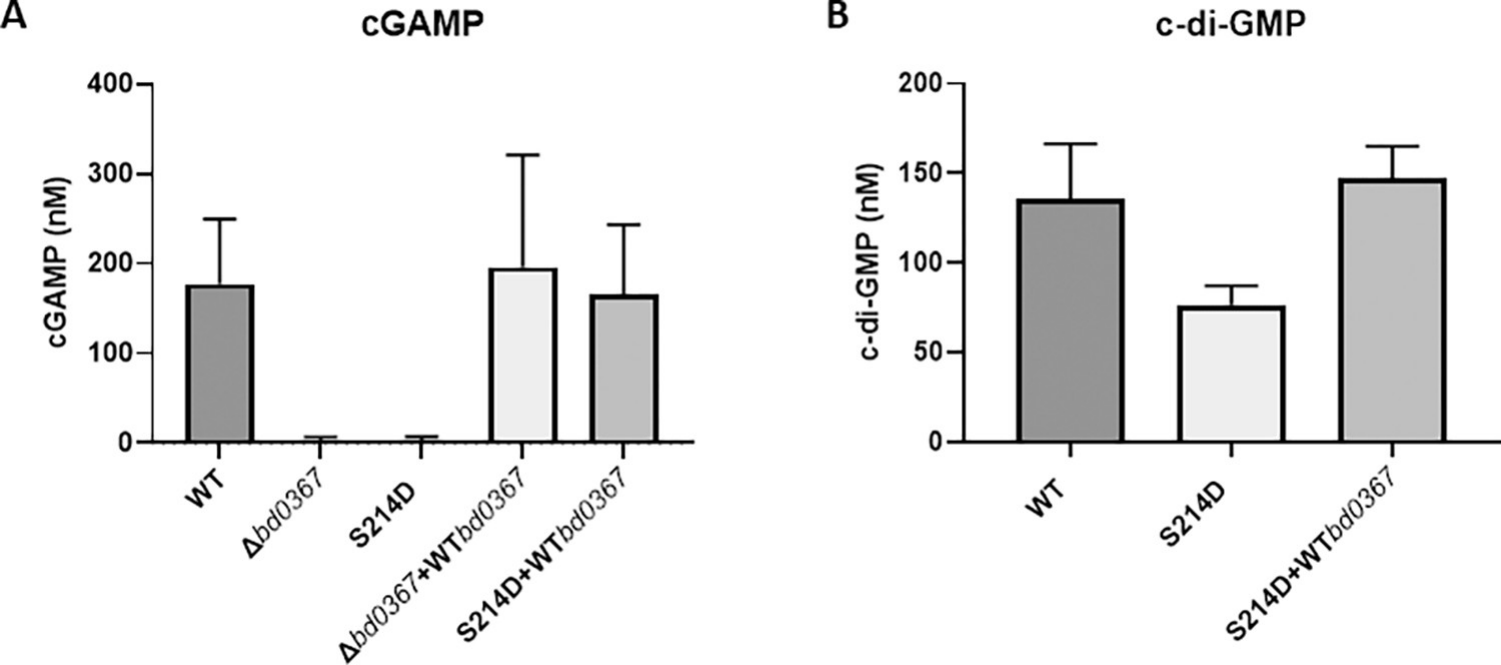
Cyclic nucleotide levels in *B*. *bacteriovorus*. A- cGAMP was extracted from whole *B*. *bacteriovorus* cells of wild type HID22 (WT), mutants *Δbd0367* and S214D, or complemented mutants (*Δbd0367*+WT*bd0367* and S214D+WT*bd0367*) and quantified using HPLC-MS/MS. Means and standard deviations of three biological replicates are presented. Cyclic GAMP levels in the complemented mutants were not significantly different from wild-type (*p* = 0.9976 for *Δbd0367*+WT*bd0367* and *p* = 0.9997 for S214D+WT*bd0367* by one-way ANOVA and Tukey’s multiple comparison test). **B- c-di-GMP** was extracted from whole *B*. *bacteriovorus* cells of wild type HID22 (WT), mutant S214D, or complemented mutant S214D+WT*bd0367* and quantified using HPLC-MS/MS. Means and standard deviations of three biological replicates are presented. Cyclic-di-GMP levels were not significantly different from wild-type (*p* = 0.0857 for S214D and *p* = 0.8326 for S214D+WT*bd0367* by one-way ANOVA and Tukey’s multiple comparison test).

Cyclic dinucleotides were extracted from the host-independent *B*. *bacteriovorus* cell cultures and analysed by LC-MS in order to investigate whether cGAMP was produced *in vivo* and to examine whether there are any differences in cyclic dinucleotide levels between the wild type strains and the *bd0367* mutant strains. Whole cell extracts from a host-independent *B*. *bacteriovorus* wild type control (HID22) was found to contain the second messenger, cGAMP, at a mean concentration of 177 nM (**Fig 3A**). Cyclic GAMP levels were completely depleted in both the *B*. *bacteriovorus bd0367* deletion and S214D site-directed mutant strains; complementation of these mutations with the wild type gene restored cGAMP concentrations to wild type levels in whole cell extracts, with mean concentrations of 196 and 166 nM respectively (**Fig 3A**). These results suggest that Bd0367 is the primary producer of cGAMP in *B*. *bacteriovorus* cells and the S214D mutant produces little to no appreciable cGAMP in cells. To investigate if the S214D mutation had any effects of c-di-GMP production, levels were compared to wild-type and the complemented S214D strain. **Fig 3B** shows that the S214D strain had slightly lower (but not statistically significantly) levels of c-di-GMP.

### Site-directed mutant *B*. *bacteriovorus bd0367*S214D strain phenocopies the *bd0367* deletion strain

As cGAMP could be detected in *B*. *bacteriovorus* whole cell extracts and levels were depleted in the Δ*bd0367* and *bd0367*S214D strains, phenotypic analysis of the mutant strains was carried out to deduce the role of this second messenger. Previously, *B*. *bacteriovorus Δbd0367* was shown to be capable of entering prey cells, but were unable to escape from the bdelloplast after replication [16]. Here, the predatory ability of the *B*. *bacteriovorus bd0367*S214D site-directed mutant strain was assessed, along with the deletion and wild type complementation strains in liquid culture. Upon mixing each of the *B*. *bacteriovorus* strains with fluorescently-tagged *E*. *coli* prey cells in liquid culture, Host-Independent (HI) cells were observed attaching to the prey cells (~0–2 hours post-mixing; **Fig D in S1 Text**). Cells of all *bd0367* genotypes (wild-type, Δ*bd0367* or *bd0367*S214D) were capable of entering prey cells and rounding them up to form a bdelloplast (~4–6 hours; **Fig D in S1 Text**). Growing *B*. *bacteriovorus* were observed microscopically within the bdelloplast as dark filaments against the fluorescent backdrop provided by the *E*. *coli* prey (**Fig D in S1 Text**). As the Δ*bd0367* strain was unable to exit prey, we tested *bd0367*S214D for this.

In culture, *B*. *bacteriovorus* HI, (prey/host-independent) cells generally grow erratically at different rates and are in different growth stages, therefore, investigation into HI strain predation is more complicated than with Host Dependent (HD) strains as they do not form synchronous infections when mixed with prey [17]. With this in mind, and the fact that the strains derived from Δ*bd0367* lack flagellar motility, additional methods were implemented to analyse the bdelloplast exit ability of the *B*. *bacteriovorus* strains.

**Fig 4:**
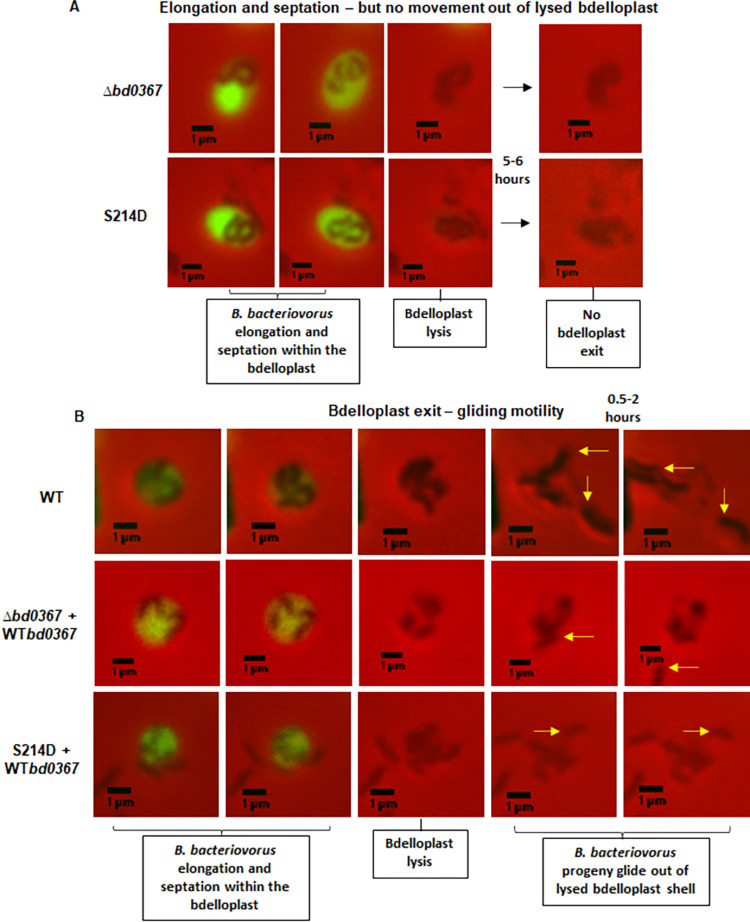
Testing the ability of *B*. *bacteriovorus* strains to exit from *E*. *coli* S17-1 prey on a hydrated 0.5% agarose pad. Stills from time-lapse microscopy are shown: **A.** The *B*. *bacteriovorus bd0367*S214D site-directed mutant strain (S214D) invaded prey, predators elongated and septated, then bdelloplast lysis occurs, seen by rapid loss of prey fluorescence. However, no motile processive exit was observed and progeny remained in the prey remnant, as was also the case for the *bd0367* deletion strain (Δ*bd0367*). **B.** Replacing the mutant *bd0367* genes with a wild-type copy gave strains of *B*. *bacteriovorus* which exited the bdelloplast by gliding motility as did the host-independent wild type strain HID13 (WT). The arrows highlight the new progeny *B*. *bacteriovorus* exiting the lysed bdelloplast. Scale bars are 1 μm. Images are representative of three biological repeats.

Post-infection of fluorescent *E*. *coli* prey with the HI *B*. *bacteriovorus* strains, time-lapse microscopy was carried out on a solid surface (0.5% agarose pad of Ca/HEPES buffer) to investigate the ability of *B*. *bacteriovorus* strains to exit prey. The *B*. *bacteriovorus bd0367*S214D site-directed mutant strain was found to elongate and septate within the prey bdelloplast, but remained immobile inside the shell of the bdelloplast (post-bdelloplast lysis, as determined by loss of *E*. *coli* prey cell fluorescence), similarly to Δ*bd0367* (**Fig 4**), even upon prolonged incubations of 12–18 hours [16]. In contrast, strains were consistently observed to exit the lysed prey bdelloplasts by gliding motility when a wild-type copy of *bd0367* replaced the mutant gene (**Fig 4**).

**Fig 5:**
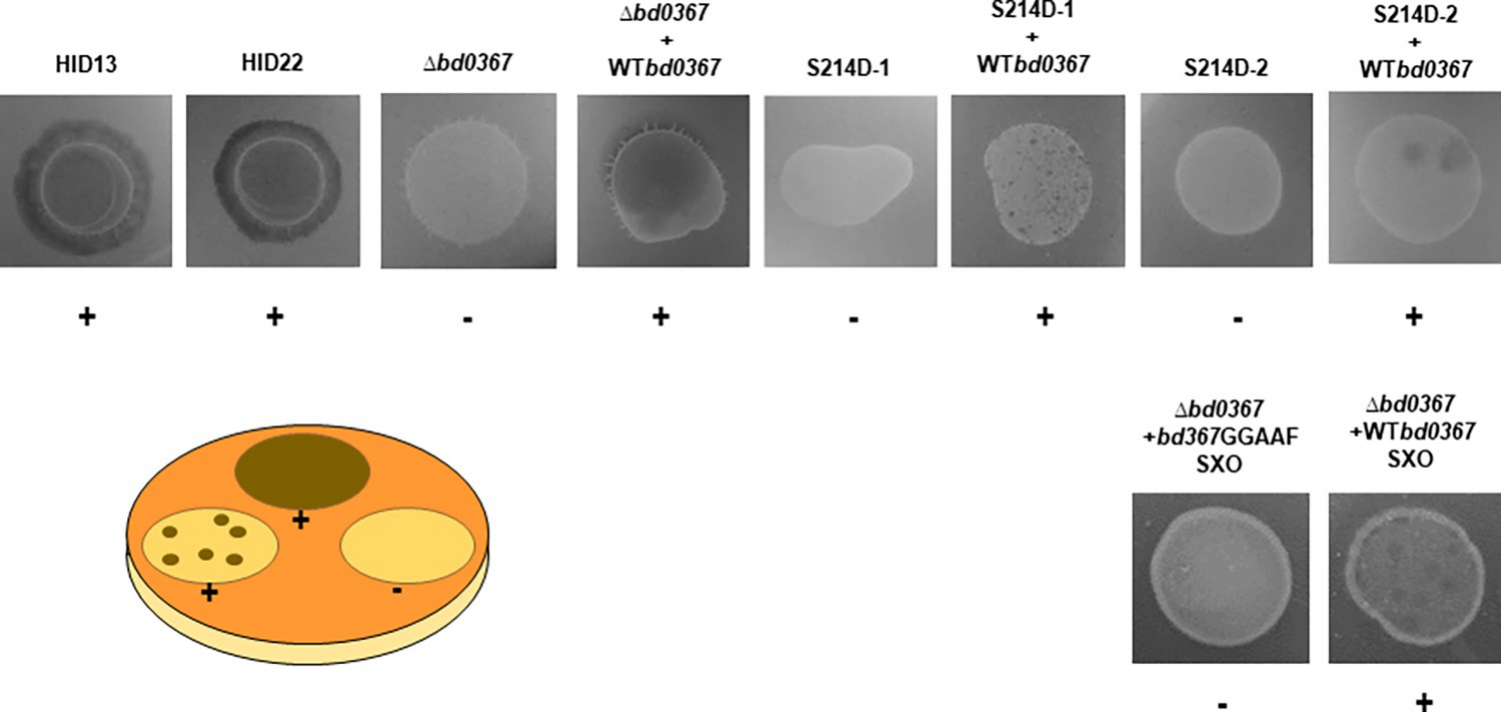
Plaque assay. Spots of host-independent (axenic) *B*. *bacteriovorus* cultures OD_600_ 1.0 on YPSC agar *E*. *coli* S17-1 prey overlay plates, incubated for 2 weeks. Two wild type host-independent *B*. *bacteriovorus* strains were tested (HID13 and HID22), alongside the *B*. *bacteriovorus Δbd0367* deletion strain, the *bd0367*(S214D) site-directed mutant (two isolates–S214D-1 and S214D-2) and the corresponding wild type complemented strains (*Δbd0367* + WT*bd0367*, S214D-1 + WT*bd0367* and S214D-2 + WT*bd0367*).). Complementation with a GGAAF mutated genotype as a single crossover was also tested (*Δbd0367* + *bd0367*GGAAF SXO) and its positive control complemented strain (*Δbd0367* + WT*bd0367* SXO). Prey killing can present itself in a number of ways in this assay, such as individual plaques, or large areas of prey clearing (**+**). Strains unable to kill prey showed a region of HI cell growth with no clearing (**-**). The numbers of experiments and plaques observed are presented in **Fig E in S1 Text**.

Plaque assays were carried out to confirm that the exit by gliding phenotypes observed via time-lapse microscopy led to completion of the predatory cycle and to the ability of the released progeny of the strains to invade more prey and to carry out some continuous predation (despite both parental defects in flagellar motility and decreased predation in HI-derived *B*. *bacteriovorus* cells). To do this the different *B*. *bacteriovorus* HI cultures were spotted onto *E*. *coli* prey lawns (prey-containing soft agar overlay plates), where they produce white regions of opacity, and predation could be seen as dark plaques or clearings within the areas of HI growth confirming whether the strains were able to exit from the bdelloplasts post-infection and go on to infect other prey cells to clear the lawn (**Fig 5**). *B*. *bacteriovorus Δbd0367* and *bd0367*(S214D) mutant strains did not produce plaques or clearings within host-independent areas of growth, compared to the extensive clearing observed for the wild type host-independent strains (HID13 or HID22), or limited clearing by the strains with the wild-type *bd0367* restored in place of the mutant copy (**Fig 5**). Rarely, a single, lone plaque was witnessed within the areas of HI growth for the *B*. *bacteriovorus bd0367*(S214D) isolates (1/13 assays for isolate one, S214D-1, and 2/14 assays for isolate two, S214D-2; **Fig E in S1 Text** for an assay summary) which we hypothesise to be suppressor mutants; however, these could never be rescued or cultured from the excess HI growth for genomic analysis.

### cGAMP production by Bd0367 is required for gliding motility

**Fig 6:**
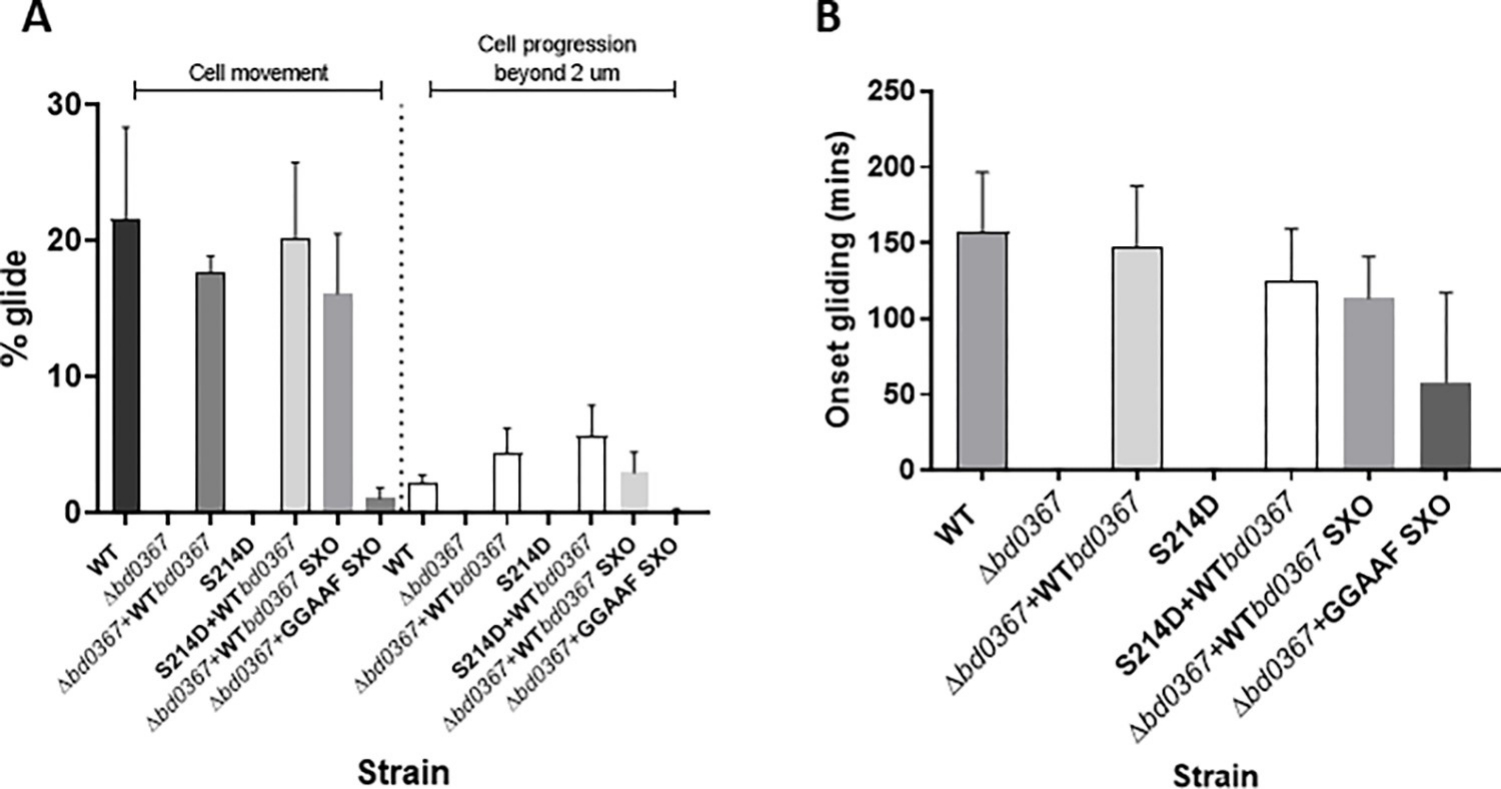
Gliding phenotype of *bd0367* mutant strains. **A.** Time-lapse microscopy was carried out over 7 hours, on a 1% agarose surface, to investigate the percentage of each *B*. *bacteriovorus* population able to glide after application from a liquid culture onto a solid surface. Two values are shown for each strain, one for the mean percentage of cells in the population that are able to move on a solid surface (any type of solid surface movement observed), and another for the mean percentage of cells that glide and progress further than 2 μm. Error bars show one standard deviation of three biological repeats. Both the *B*. *bacteriovorus bd0367* deletion (*Δbd0367*) and *bd0367*(S214D) site-directed mutant strains were unable to glide. There was no significant difference in the gliding abilities of the strains that are able to glide (one-way ANOVA with Tukey’s multiple comparisons test–Cell movement: HID13 vs *Δbd0367+*WT*bd0367*, *p* = 0.64; HID13 vs *bd0367*(S214D)+WT*bd0367*, *p* = 0.94; *Δbd0367*+WT*bd0367* vs *bd0367*(S214D)+WT*bd0367*, *p* = 0.8. Cell progression beyond 2μm: HID13 vs *Δbd0367+*WT*bd0367*, *p* = 0.35; HID13 vs *bd0367*(S214D)+WT*bd0367*, *p* = 0.08; *Δbd0367*+WT*bd0367* vs *bd0367*(S214D)+WT*bd0367*, *p* = 0.62). Gliding of the GGAAF complemented strain (*Δbd0367*+GGAAF SXO) was significantly less (*p* = 0.0044 for cell movement, *p* = 0.0327 for cell progression by unpaired *t*-test) than the control single crossover strain (*Δbd0367*+WT*bd0367* SXO). **B.** The time taken for each strain to initiate gliding (onset of gliding) over the 7 hour time-lapse. The mean onset of gliding (± standard deviation, 3 biological repeats) of the complementation strains were found to not significantly differ (*Δbd0367*+WT*bd0367* = 2.47±0.66 hrs; *bd0367*(S214D)+WT*bd0367* = 2.08±0.57 hrs) to the host-independent wild type strain gliding onset (HID13 = 2.62±0.6 hrs) (one-way ANOVA with Tukey’s multiple comparisons test: HID13 vs *Δbd0367*+WT*bd0367*, *p* = 0.95; HID13 vs *bd0367*(S214D)+WT*bd0367*, *p* = 0.53; *Δbd0367*+WT*bd0367* vs *bd0367*(S214D)+WT*bd0367*, *p* = 0.71). Onset of gliding was not significantly different (*p* = 0.21 by unpaired *t*-test) for the GGAAF complemented strain (*Δbd0367*+GGAAF SXO) compared to the control single crossover strain (*Δbd0367*+WT*bd0367* SXO).

As exit of the newly replicated progeny of *B*. *bacteriovorus* wild type and complementation strains appeared to be by gliding motility from depleted bdelloplasts on solid surfaces, the *bd0367*(S214D) strain was tested outside prey and found to be unable to glide on a solid agarose surface, as was the Δ*bd0367* deletion strain (0% of cells observed; **Fig 6**). The inability to glide could be rescued by complementation with the wild type gene, with strains displaying gliding behaviours comparable to the wild type host-independent strain, HID13, when wild-type *bd0367* was restored in place of the mutant gene (**Fig 6**). Within 8 hours of being applied to a 1% agarose surface, 17.7% of the *Δbd0367*+WT*bd0367* cells were observed to glide (with 4.35% of the population progressing further than 2 μm), with an average gliding onset of 2.5 hours (**Fig 6**). Similarly, 20.28% of the *bd0367*(S214D)+WT*bd0367* cells were able to glide (5.678% progressing), with an average gliding onset of 2.1 hours (**Fig 6**). These data did not significantly differ to data for wild type analyses (one-way ANOVA with Tukey’s multiple comparisons test) where 21.59% of the population was able to glide, with an average gliding onset of 2.6 hours (2.17% of the population progressing further than 2 μm) (**Fig 6**). Complementing the *Δbd0367* strain with an enzymatically c-di-GMP null mutant *bd0367*GGAAF drastically reduced gliding relative to complementing with the wild-type *bd0367*, with only 1.07% of the population able to glide and 0.12% progressing (Fig 6).

**Fig 7:**
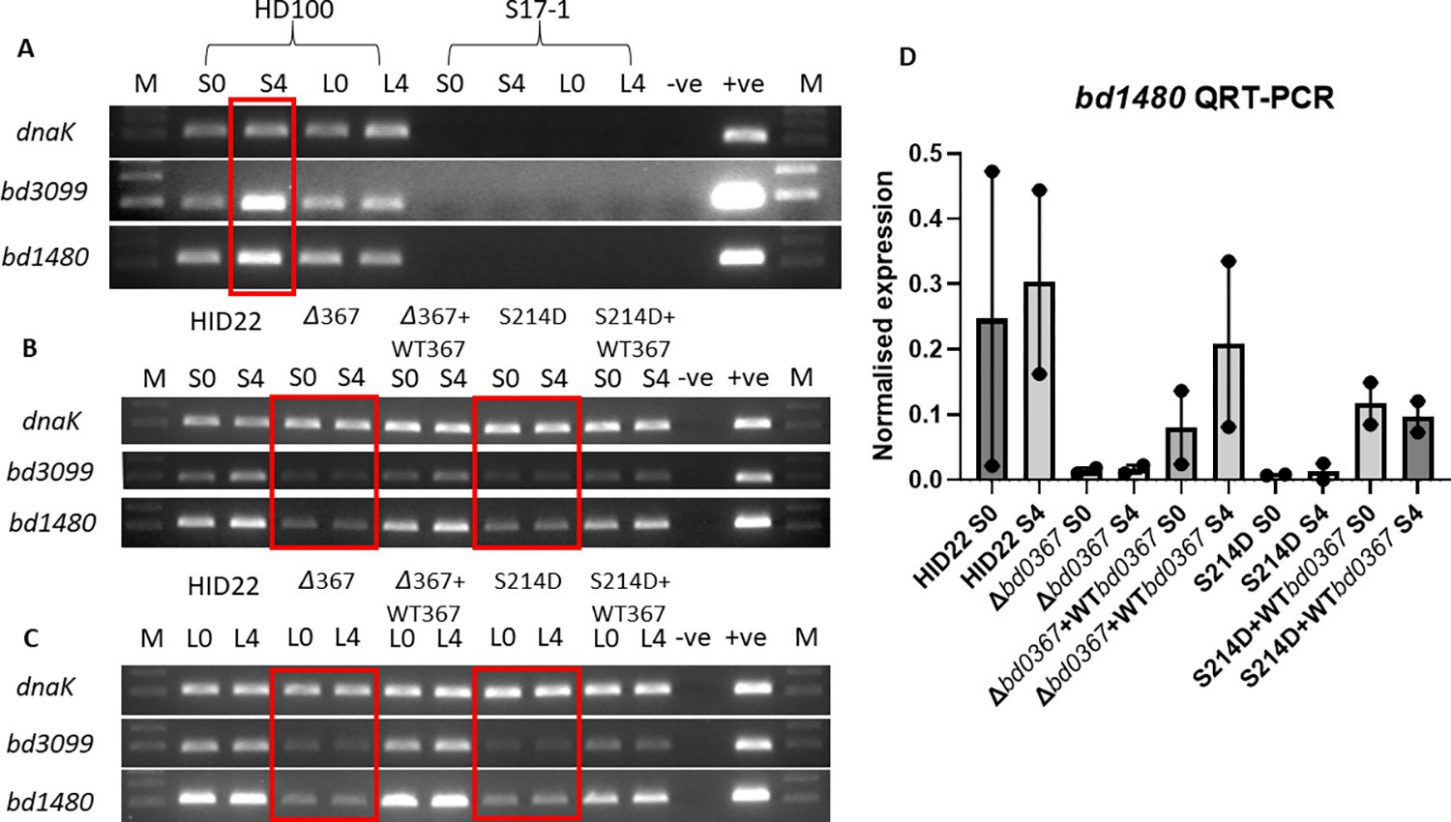
RT-PCR investigating gliding gene expression on a surface or in liquid. **A-** Expression of gliding genes *bd3099* and *bd1480* and control housekeeping gene *dnaK* in wild-type, HD grown, predatory *B*. *bacteriovorus* HD100 incubated on a surface for 0 (S0) or 4 hours (S4) and controls incubated in liquid (L0 and L4) show induction of the gliding genes, but not control gene *dnaK* at 4 hours on surface (red box). Control RNA isolated from the prey *E*. *coli* cells (S17-1) did not give products with these primers designed to specifically anneal to the *Bdellovibrio* genes. **B-** RT-PCR with RNA isolated from host independent (HI) strains incubated on a surface for 0 (S0) or 4 hours (S4) shows lower expression of predicted gliding genes in mutant strains (Δ367 and S214D) relative to wild type (HID22) and reconstructed wild-type *bd0367* strains (+WT367; red boxes), but similar expression of control *dnaK* gene in all strains. **C-** HI cultured strains incubated in liquid for 0 (L0) or 4 hours (L4) also show lower expression of predicted gliding genes in mutant strains (Δ367 and S214D) relative to wild type (HID22) and reconstructed wild-type *bd0367* strains (+WT367; red boxes), but similar expression of control *dnaK* gene. Images are representative of two independent repeats.–ve no template negative control, +ve genomic DNA positive control, M- NEB 100bp ladder; lower band 100bp, higher band 200bp. **D-** Quantitative RT-PCR to confirm expression pattern of *bd1480*. Quantification of cDNA was by comparison to purified PCR product standard curve and normalized relative to whole signal from each biological repeat.

To investigate how cGAMP synthesised by Bd0367 may regulate gliding motility, we tested the expression of putative gliding motility genes by RT-PCR. The genome sequence of *B*. *bacteriovorus* HD100 revealed four complete operons and two smaller operons annotated as gliding motility related [27]. Gliding motility is the product of many proteins expressed across the whole envelope of delta proteobacterial cells from the inner membrane to the surface of the outer membrane. Gliding has been extensively functionally studied in myxobacteria with important gliding genes noted as evolutionarily conserved in *Bdellovibrio* [28–30]. By isolating total RNA from wild-type *Bdellovibrio bacteriovorus* HD100, applied to and incubated on a surface, we identified two genes from these gliding gene clusters. These are *bd3099*, a conserved Von Willebrand Adhesin motif encoding gene, and *bd1480*, an *aglQ* gliding motility gene that is predicted to encode a component of the transmembrane proton conducting complex required for gliding engines in other delta proteobacteria. Both genes were found to be upregulated after 4 hours of *B*. *bacteriovorus* adaptation to a surface (S4) compared to time 0 (S0) and liquid (LO, L4) exposure controls (**Fig 7A**). This suggested that the products of these genes are involved in the production of the protein engines required for gliding motility on surfaces.

Testing the expression levels of these gliding genes after surface incubation of the HI cultures of the Bd0367 mutants shows that both gliding genes were expressed at a higher level in wild-type and wild-type *bd0367* reconstructed strains compared to both the *Δbd0367* and the *bd0367*(S214D) strains (**Fig 7B**). Both *bd3099* and *bd1480* genes were also expressed in liquid controls for these HI cultures. Due to the expression of the genes in liquid there was also transcription seen at time zero on the surface for these HI cultures (SO) (**Fig 7C**). (This expression in liquid of gliding genes by wild-type HI cells is probably due to cellular touching/sensing, which induces surface sensing and, therefore, gliding in the HI cultures, as opposed to the highly separated motile flagellate HD100 cells where it is only induced on a surface). However, clear differences in *bd3099* and *bd1480* expression in the *Δbd0367* and *bd0367*(S214D) mutants versus wild type or wild-type *bd0367* reconstructed strains could be seen in both conditions (**Fig 7B–7C**). The relative expression levels of these gliding operons in HI cells compared to HD cells is in agreement with microarray [31] and RNA-seq experiments [32]. The lower levels of expression of *bd3099* and *bd1480* in the mutant *Δbd0367* and *bd0367*(S214D) strains suggest that Bd0367-synthesised cGAMP may regulate the transcription of these gliding genes (or alternatively, destabilise the mRNA post-transcriptionally).

### *fliS* genotyping of mutants implicates a role of Bd0367 c-di-GMP production in flagellar swimming motility

All strains studied here were derived from the original *bd0367* deletion strain (Δ*bd0367*) and were unable to swim in liquid culture, a phenotype originally described for the *bd0367* deletion strain. This could not be complemented with *bd0367-mCherry* in a merodiploid strain [16] and here, we observed that all strains derived from this Δ*bd0367* deletion strain also remain non-motile, even when the mutated *bd0367* (deletion or S214D variant) was replaced with wild-type *bd0367*.

To understand why, whole genome sequencing was carried out on the original Δ*bd0367* strain and revealed a point mutation within the flagellin specific chaperone gene, *fliS* (*bd0612*). The mutation at codon 39, AAG (originally a lysine residue) to TAG (a stop codon), prevents the majority of the chaperone FliS being translated and as this protein is required to chaperone and thus organize the productive export of FliC flagellin proteins to assemble a flagellar filament in other bacteria [33] is therefore most likely responsible for the lack of swimming motility observed in *B*. *bacteriovorus Δbd0367* and all strains derived from this.

**Fig 8:**
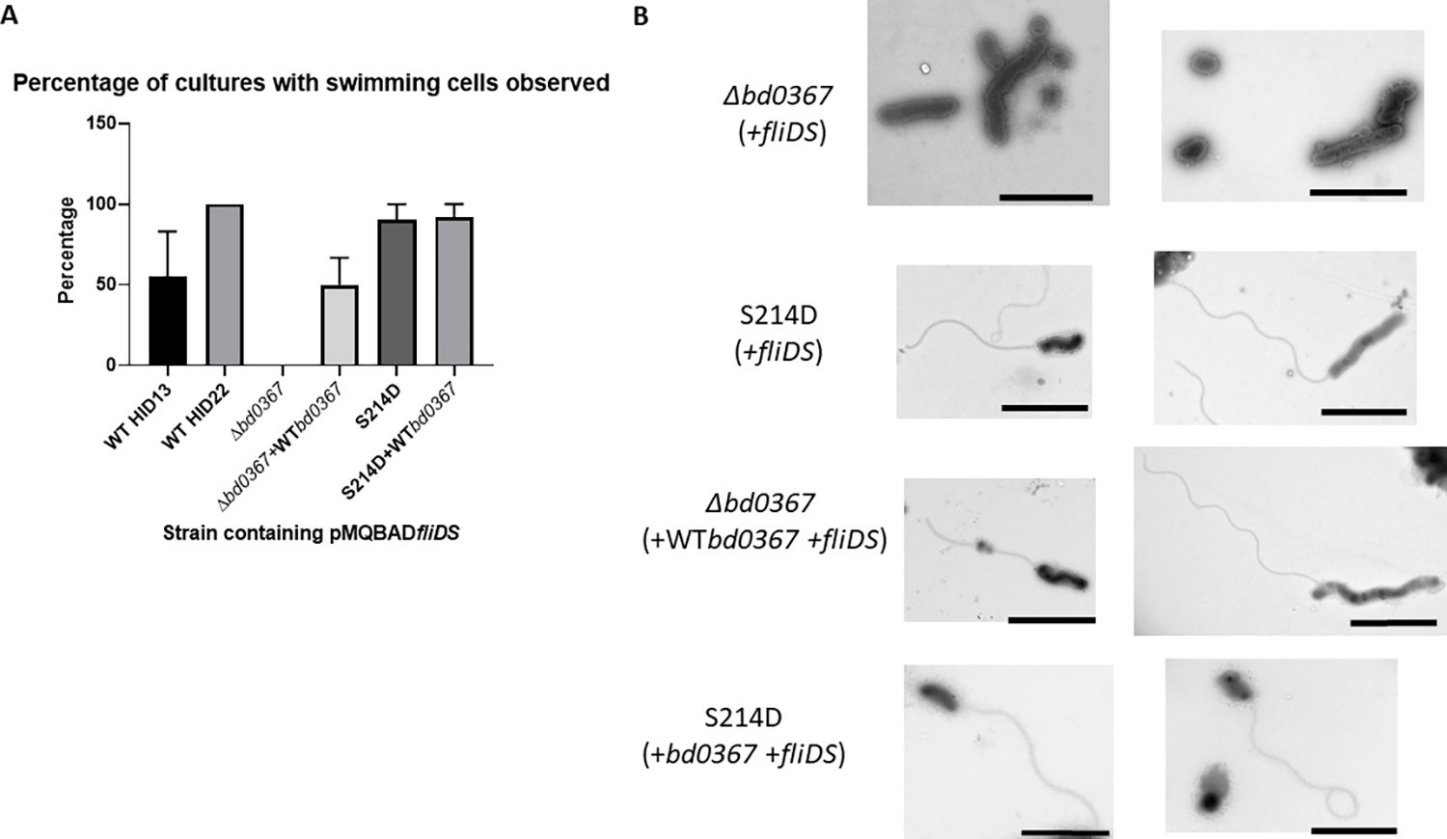
Swimming phenotype of all strains complemented with *fliDS in trans*. **A-** Cultures were grown in PY broth supplemented with gentamycin and scored for presence or absence of actively swimming cells. No swimming cells of the *Δbd0367* strain were ever observed, whilst all other strains did exhibit swimming. Data are from 3 biological repeats with cultures grown above OD_600_ 0.45 per repeat (dependent upon variable growth phenotype of the HI cells and cutoff for this OD_600_ chosen as swimming cells were never observed in cultures of OD_600_ < 0.45; see Table C in S1 Text for *n* values). Error bars are SEM. **B-** Transmission electron micrographs of cells grown after complementation with *fliS* supplied *in trans* on a plasmid. No flagella were seen on Δ*bd0367* cells, but flagella were seen on cells of the *bd0367*(S214D) strain and the strains reconstructed with wild-type *bd0367* (when also complemented with *fliS*). Cells were stained with 0.5% uranyl acetate for 1 minute on carbon formvar grids and imaged on a FEI Technai bio-twin 12 TEM. Scale bars are 2 μm.

To test this, we complemented this mutation in control (wild-type *bd0367*) and mutant *bd0367* strains by supplying *fliDS* with flanking DNA *in trans* on a plasmid pMQBAD (as *fliS* is predicted to be in an operon driven by a promoter of the upstream gene *fliD*, *bd0611*). Exconjugants of the Δ*bd0367* strain grew poorly in liquid PY broth supplemented with gentamycin to select for upkeep of the *fliDS*-encoding plasmid. After several rounds of subculturing on PY agar plates containing gentamycin, this growth defect of Δ*bd0367* strain in the presence of the *fliDS*-encoding plasmid was overcome, and we purified plasmid DNA, suspecting a mutational adaptation (see next paragraph below). All cultures were scored for swimming by phase contrast microscopy. Whilst HI cells generally exhibit variable morphology and motility, actively swimming cells were consistently seen in wild type Bd0367 control strains (**Fig 8A;** HID13 and HID22), in strains with restored wild-type *bd0367* at the mutant *bd0367* locus (Δ*bd0367*+WT*bd0367* and *bd0367*(S214D)+WT*bd0367*) and in the *bd0367*S214D mutant strain. These actively swimming cells were never observed in the *Δbd0367* mutant, even though it had adapted to grow more rapidly. Consistent with this, transmission electron microscopy revealed examples of flagellated cells in swimming motile strains whilst only cells lacking flagella were observed for the Δ*bd0367* strain (**Fig 8B**).

Sequencing of the introduced plasmid showed that in all cases (5/5; **Table C in S1 Text**), the *fliS* gene introduced to the Δ*bd0367* strain had acquired the same stop codon mutation observed in the genome of this strain, accounting for the lack of observed swimming cells. This mutation also arose in the plasmid-borne *fliS* gene in the other strains (discussed in **Supplementary Discussion in S1 Text**), but unlike the Δ*bd0367* strain the wild-type *fliS* sequence could be detected in the *bd0367*S214D mutant strain. The sequence was determined by PCR amplification of the plasmid sequence (as plasmid extraction from *Bdellovibrio* does not give a high enough yield for Sanger sequencing), so the different sequences obtained likely represent a mixed population of wild-type and mutant sequences for these strains.

Together with the observation of swimming and flagellated cells, the data suggest that wild-type *fliS* cannot be tolerated in the Δ*bd0367* strain, but can be tolerated in the *bd0367*(S214D) strain. Since the S214D strain is defective in cGAMP synthesis activity but not c-di-GMP synthesis, the fact that it phenocopies the WT and complemented strains shows that the effect of bd0367 deletion on flagellar biogenesis is not due to cGAMP production. Instead, the situation implies a role for regulation of flagellar biogenesis by c-di-GMP production by Bd0367.

To test if this regulation was feeding back to affect flagellar structural gene transcription, we monitored levels of expression of motility related genes by RT-PCR. Levels of expression of *fliF* and *fliC5* as well as control gene *dnaK* and of *cstA* (a transcriptional regulatory protein known to be *fliS-*associated in *Bacillus*), were at similar levels for *bd0367* mutants and restored wild-type *bd0367* strains in cultures incubated in liquid (**Fig F in S1 Text**) suggesting that control of swimming motility by Bd0367 is not at the transcriptional level.

## Discussion

As cGAMP signalling in bacteria is a relatively recent discovery, little is known about its role and so this signalling has only been linked to a handful of bacterial phenotypes thus far. cGAMP has been shown to regulate genes related to exoelectrogenesis in *Geobacter* [8], motility and biofilm formation in *E*. *coli* [34] and chemotaxis and fatty metabolism genes in *Vibrio* [5], the latter affecting intestinal colonisation of mice. It has also recently been shown to function in protection against phage [35] and may play many other important roles.

Previous work [10] discovered a subset of GGDEF proteins, named Hypr GGDEF proteins, that are able to synthesise c-di-GMP, cGAMP, and c-di-AMP *in vitro* and regulate cGAMP signaling *in vivo* [11] and one of these proteins was identified in *B*. *bacteriovorus* HD100, Bd0367. Here we demonstrate *in vivo* that Bd0367 is the primary producer of cGAMP in this predatory bacterium (**Fig 3**). A serine residue (S214) is present within the GGDEF domain of Bd0367, whereas canonical GGDEF domain proteins contain an aspartate residue at this position [10,12]; this residue was confirmed as the specificity residue, allowing this enzyme to produce primarily cGAMP, with a Bd0367(S214D) variant producing almost exclusively c-di-GMP (**Fig 2 and Fig C in S1 Text**). *In vivo* the S214D mutation produced a host-independent *B*. *bacteriovorus* strain with no detectable cGAMP production that behaved in a comparable manner to the *B*. *bacteriovorus Δbd0367* strain previously described [16]; unable to glide on a solid surface, it could only be isolated host-independently. If offered to a prey cell, Bd0367(S214D) could invade to begin predation, but could not escape from the prey cell remnants to continue the predatory cycle (via processive gliding motility on a solid surface). These phenotypes were previously attributed to Bd0367 c-di-GMP signalling being disrupted, however, it is now clear that cGAMP signalling is controlling this processive gliding motility as the Bd0367(S214D) mutant (still capable of producing c-di-GMP; **Fig 2 and Fig C in S1 Text**) was equally deficient in gliding motility (**Figs 4–6**). An enzymatically c-di-GMP null mutant, with the active site mutated from GGDEF to GGAAF, was unable to complement the deletion mutant; unable to form predatory plaques and with greatly disrupted progressive gliding ability. The fact that there was a small amount of initiation of gliding observed with the GGAAF mutant, as compared to none seen in the absence of *bd0367* or in the S214D mutant suggests that the presence of the Bd0367 protein is required for regulating gliding and this processive gliding is induced by cGAMP production. It is possible that the balance between production of the different cyclic nucleotides by Bd0367 induces protein; protein interactions to regulate the different motilities.

The Δ*bd0367* mutant and restored wild-type *bd0367* strains in this study were derived from the original Δ*bd0367* strain isolated by Hobley *et al*. [16] that was noted at the time to be defective in flagellar swimming motility. Here, we discover that this is due to a mutation within the flagellar chaperone *fliS*, as complementation of the strains with wild-type *fliS* restored motility in all but the Δ*bd0367* strain. The Δ*bd0367* strain, however, grew slowly in a prey-independent HI liquid culture until the added *fliS* gene acquired a severely truncating mutation. FliS is a chaperone that binds flagellin monomers co-ordinating flagellin production to flagellin export and flagellar filament assembly for swimming motility in *Salmonella* [33]. It could be that in *B*. *bacteriovorus* the WT FliS chaperone was capable of also allowing non-beneficial export, through the hollow centre of the growing flagellum, of a molecule needed by the Δ*bd0367* strain for growth/viability. Transcriptional studies showed that the *fliF* gene, which encodes the protein that forms the centre of the hollow core of the flagellum through which external flagellar components are normally exported, was being expressed normally in the Δ*bd0367* strain, so deleterious chaperone effects are possible but investigating this mechanism needs a larger study beyond the scope of this work.

The apparent incompatibility of a wild-type *fliS* gene or need for a truncated *fliS* gene in the Δ*bd0367* strain implicates a role for Bd0367 c-di-GMP production in cross talk to the biosynthesis of flagella or the physiological consequences of flagellar motility itself. Surprisingly, the Bd0367(S214D) strain, which still produces c-di-GMP but not cGAMP, was compatible with a wild-type *fliS* gene. Therefore, these results together show clear differentiation in the roles of c-di-GMP and cGAMP produced by the same enzyme.

To our knowledge, this is the first demonstration that a single GGDEF enzyme has dual functions *in vivo* that are assignable to its production of two distinct cyclic dinucleotide signals. The dual roles of Bd0367 in *B*. *bacteriovorus* contrasts with recent observations [11] in *G*. *sulfurreducens* that GacA (the *Geobacter* homologue of Bd0367) selectively affects cGAMP but not c-di-GMP levels and controls extracellular electron transfer to Fe(III) oxides but not to electrodes. Electrogenesis through electrodes instead requires c-di-GMP signaling through the canonical diguanylate cyclase EsnD. Taken together, these studies reveal that the subset of GGDEF enzymes known as Hypr GGDEFs can serve either singular or multiple signalling roles, depending on the needs of the organism. Notably, *G*. *sulfurreducens* has 29 GGDEF enzymes in total, whereas the predatory *B*. *bacteriovorus* has 4, including Bd0367.

Hallberg, Chan, *et al*. [11] also found that which cyclic dinucleotide is produced by GacA is influenced not just by the S214-equivalent amino-acid GacA S347; but also by a newly identified cross-dimer residue that sits above the nucleotide substrate. Canonical GGDEFs that produce c-di-GMP harbour an arginine at this position, whereas GacA from *G*. *sulfurreducens* has a tyrosine. The Y304R mutant of GacA in fact produces both cGAMP and c-di-GMP. Intriguingly, Bd0367 harbours a methionine at this position.

The timing and mechanism(s) of activation of Bd0367 to synthesise c-di-GMP and cGAMP in the predatory life cycle of *B*. *bacteriovorus* are currently under study and what triggers cGAMP production vs c-di-GMP production, and in what conditions, is particularly intriguing. We show here (**Fig 3**) that Bd0367 produces cGAMP *in vivo* and have previously demonstrated that c-di-GMP levels in a *bd0367* deletion strain dropped compared to the wild type strain (c-di-GMP was still detected, however, due to the presence of 3 other functional GGDEF proteins [16]) suggesting that both of these Bd0367-synthesised secondary messengers are important in *Bdellovibrio*. There are many parts of the predatory process to regulate–hunting prey, attachment, entry, intrabacterial growth and replication, sensing self and prey in the enclosed bdelloplast environment, signalling to escape, deciding which motility apparatus to induce dependent on liquid or semi-dry surface environments sensed, and inducing the physical act of leaving the exhausted prey cell. The action of multiple second messenger signalling cascades is possibly required for such a complex series of events to take place. Cyclic-di-GMP synthesised by Bd0742 DgcB and Bd1434 DgcC have been found previously to be involved in regulating separately predatory invasion of prey or free-living lifestyles [16,25]. Here we show that cGAMP synthesised by Bd0367 is involved in prey escape through gliding motility and that cyclic di-GMP synthesised by Bd0367 is likely involved in flagellar motility prey escape. Hallberg *et al*. [10] demonstrated that the capability of *Geobacter sulfurreducens* GacA to produce cGAMP versus c-di-GMP depends on the relative levels of ATP/GTP under physiological conditions and with c-di-GMP levels inhibiting cGAMP production via I-site inhibition. *B*. *bacteriovorus* is unique among cGAMP utilizing bacteria analysed to date in that it resides within a prey host, so it may be exposed to drastically different ATP/GTP levels depending on host lysis status. Bd0367 also contains an N-terminal receiver (REC) domain with a canonical phosphorylation site (D74), and therefore may be part of a two-component system, the kinase of which has yet to be identified. It is possible that (ATP-dependent) phosphorylation causes product identity to switch (instead of simply activating the enzyme); intriguingly, we found that the D74E phosphomimic mutant has a product distribution shifted in favour of c-di-GMP over cGAMP, although this mutant overall is less active, which may be due to either regulation or destabilization (**Fig C in S1 Text**). We also have previously speculated that single versus doubly phosphorylated states could have different activities [11]; or different Rec activation mechanisms could give rise to different signals. The many tiers of enzyme regulation, product distribution, and phenotypic response remains an attractive road for future inquiry.

Alternatively, regulation of which signal is being sensed could be dictated by specific phosphodiesterases that are associated with Bd0367. Recently, Bd0367 has been shown to interact with Bd1971, a c-di-GMP degrading protein that integrates c-di-GMP and cAMP signals in *B*. *bacteriovorus* [36] so these proteins may be an important hub integrating secondary messaging signals. Bd2325 was recently identified to have cGAMP-specific phosphodiesterase activity that requires either Mn^2+^ or Ni^2+^ [37] and is expressed at higher levels in attack phase compared to either intraperiplasmic growth phase or host-independent growth [31,32], potentially resetting cGAMP levels after prey exit has been signalled by this molecule.

Here, we show (**Fig 7**) downstream transcriptional regulation of the gliding motility genes by cGAMP-producing Bd0367, as mutants of Bd0367 have lower levels of transcription of two such putative genes. However, regulation of flagellar motility may be at translation or export and not be at the transcriptional level; as genes tested do not seem to be affected by mutational status of *bd0367* and the FliS involvement is a post-translational interaction with FliC flagellar filament proteins. As *Bdellovibrio* produce a flagellum just prior to cell division at the end of their intracellular growth cycle, sensing of the junction of these processes are likely linked, and indeed, the flagellar master regulator FliA has evolved in *Bdellovibrio* to control expression of a large regulon of predatory attack phase, and not just flagellar, genes [38]. Bd0367 c-di-GMP production likely interfaces with this attack phase preparation and flagellar development system, which may include the FliS chaperone. HI cultures of *B*. *bacteriovorus* are asynchronous and have a cell density dependence for growth, possibly mimicking some of the close quarters contact of predators growing inside the prey bdelloplast [17]. Thus the absence of this normal regulatory interface (by deletion of *bd0367*) may have been lethal, and halting FliS chaperone activity may have stopped the export of a factor needed for developmental cues at the end of growth. The role of the Hypr GGDEF protein Bd0367 is likely therefore to integrate signals detecting the surface (semi-dry) versus planktonic (liquid) location of the bdelloplast at the end of *Bdellovibrio* growth (possibly including the cell contact status of predators inside prey which is also expressed partially in dense HI cultures) and transmitting these in the form of c-di-GMP versus cGAMP to choose the appropriate motility apparatus to develop in the dividing and newly-forming attack-phase cells. The fact that bdelloplast lysis was observed in the Bd0367 mutants, but that the *Bdellovibrio* did not glide processively to emerge from the bdelloplast, suggests that Bd0367 is a check point in development that detects liquid or surface and participates in transducing this into choice of an appropriate motility system, thus exit is stalled until this has effectively completed. While a much more extensive programme of work is required to verify the full mechanisms of this regulation, the link to flagellar or gliding motility induction is shown by our results.

Here we show that a single protein can produce at least two secondary messengers from the same active site and demonstrate, to our knowledge for the first time, that a synthase with these dual activities is exploited for dual signaling functions *in vivo*. Bd0367 produces distinct signals to regulate two separate motility systems- gliding or flagellar- either of which is essential for completion of bacterial predation by movement out of prey, completing the predatory life cycle of *B*. *bacteriovorus*.

## Methods

### Bacterial strains, plasmids and growth conditions

*Bdellovibrio* and *E*. *coli* strains and plasmids in this study are listed in **Tables A** and **B in S1 Text** respectively. Host-Independent (HI) *B*. *bacteriovorus* strains were grown on PY agar or PY liquid medium (200 rpm shaking) at 29°C free of prey cells. If *E*. *coli* cells were required for predatory assays, either *E*. *coli* S17-1 or fluorescent *E*. *coli* S17-1::pMAL-p2_mCherry [39] were used, grown in YT medium at 37°C, 200 rpm shaking. Plasmids were maintained in fluorescent *E*. *coli* with 50 μg ml^-1^ of ampicillin and IPTG (isopropyl-β-D-1-thiogalactopyranoside) being added to media for induction of fluorescent strains at 200 μg ml^-1^.

### Generation of *B*. *bacteriovorus* site-directed mutant and complementation strains

The *B*. *bacteriovorus* HD100 site-directed mutant strain *bd0367*(S214D) and restored wild-type *bd0367* strains (where the *Δbd0367* and *bd0367*(S214D) mutant loci were replaced with the wild type *bd0367* gene) were made similarly to how markerless deletions are generated in *B*. *bacteriovorus* (as previously described [16,40] adapted from Steyert & Pineiro [41]). PCR was used to generate either *bd0367* wild type or mutant (S214D) gene fragments with 1 kb flanking the gene (primers can be found in **Table B in S1 Text**). The NEBuilder HiFi DNA Assembly kit (NEB), based on Gibson assembly cloning [42], was used to assemble the *bd0367* gene fragments, including the flanking regions, into the suicide vector pK18*mobsacB* (which was digested with EcoRI and BamHI prior to assembly). The resulting plasmid was conjugated into the required *B*. *bacteriovorus* host-independent strain via an *E*. *coli* S17-1 donor strain. The resulting merodiploid strain (plasmid integrated into the *B*. *bacteriovorus* genome via a single cross-over event within the *bd0367* gene or the flanking regions depending on which strain targeted) was then grown in the presence of sucrose to induce a second recombination event. This allowed the site-directed mutant strain to be generated by inserting *bd0367*(S214D) into the *B*. *bacteriovorus* Δ*bd0367* genome. Restored wild-type *bd0367* strains were generated by inserting the wild type gene into the *B*. *bacteriovorus* Δ*bd0367* genome and by fully replacing the site-directed mutant gene with the wild type version in the *B*. *bacteriovorus bd0367*(S214D) genome (the second recombination event fully replaced the mutant gene). Testing complementation of the Δ*bd0367* strain with the *bd0367*(GGAAF) was achieved by mutating the wild-type complement plasmid with a Q5 site-directed mutation kit (NEB) according to manufacturer’s instructions to change the sequence encoding the GGDEF motif from GGCGGGGATGAATTC to GGCGGGGCCGCCTTC (encoding GGAAF). This new plasmid and control plasmid (with wild-type *bd0367*) were conjugated into *B*. *bacteriovorus* and maintained as single-crossover exconjugants with kanamycin selection.

### Phase contrast and fluorescent microscopy

Time-lapse microscopy was carried out on a Nikon Eclipse E600 epifluorescence microscope equipped with a 100x objective lens, hcRED filter where necessary (excitation 560 to 600 nm; emission 610 to 665 nm) and a Hamamatsu Orca ER camera.

Phase contrast and fluorescent microscopy was also carried out on a Nikon Ti-E microscope with was equipped with a Plan Apo 60x/1.40 Oil Ph3 DM objective, 1.5x intermediate magnification, a Cy5 filter cube and an Andor Neo sCMOS camera, using the mCherry settings for detection of mCherry tagged proteins (excitation 560 to 600 nm; emission 610 to 665 nm) where necessary.

Images and videos were analysed with ImageJ and the MicrobeJ plug-in [43].

### Bdelloplast exit assays

In order to start *B*. *bacteriovorus*—*E*. *coli* infections, 1 ml of the desired *B*. *bacteriovorus* host-independent strain at an OD_600_ of 1.0 in CaHEPES (originally grown in PY media before being pelleted and resuspended in CaHEPES) was mixed with 1.5 ml of fluorescently tagged *E*. *coli* S17-1::pMAL-p2_mCherry prey at an OD_600_ of 1.0 in CaHEPES (originally grown in YT media containing IPTG at a final concentration of 200 μg/ml). Infections were incubated for 2.5 hours at 29°C with shaking at 200 rpm before 10 μl was transferred onto a 0.5% agarose surface (on top of a microscope slide, containing wells for hydration) and incubated for a further 2.5 hours at 29°C in a humid environment.

This extensive incubation of host-independent *B*. *bacteriovorus* cells with the *E*. *coli* prey in both liquid and solid surface environments allowed the host-independent strains, which do not form synchronous infections like host-dependent strains, to meet the prey cells (in some cases the predators were non-motile and therefore agitation in liquid helped with encounters) and then acclimatise to the solid surface before exit abilities were assessed.

*B*. *bacteriovorus* bdelloplast exit ability was then assessed via time-lapse microscopy on the Nikon Eclipse E600 epifluorescence microscope (on the 0.5% hydrated agarose pad). Time-lapse microscopy was run up to 18 hours with 150 second intervals between frames.

### Gliding motility assay

*B*. *bacteriovorus* gliding motility was assessed via time-lapse microscopy on the Nikon Eclipse E600 epifluorescence microscope. *B*. *bacteriovorus* cells from 100 μl of host-independent culture were concentrated 10-fold in CaHEPES and applied to a 1% agarose:CaHEPES surface on a microscope slide (containing wells to allow hydration). Time-lapse microscopy was run over the course of 7 hours with 150 second intervals between frames.

Resulting videos were then analysed using ImageJ using the MicrobeJ plugin to define individual *B*. *bacteriovorus* cells, using the following parameters: area 0–1.5 μm^2^, length 0.5–5 μm, width 0.2–0.8 μm, and all other parameters were set as default. The image was then manually inspected to confirm that the majority of the cells had been correctly identified and the ImageJ cell counter tool was used to manually inspect cells to determine onset of gliding and percentage of cells gliding within a population. Three biological replicates were analysed for each strain.

### Plaque assays

*B*. *bacteriovorus* HI strains were grown in PY media and diluted to OD_600_ 1.0, 10 μl of this culture was then spotted onto a YPSC prey overlay plate (0.6% YPSC agar containing S17-1 prey cells poured over a 1% YPSC agar layer) and allowed to dry before being incubated at 29°C for 2 weeks. Regions of HI growth were visually inspected for prey killing (plaques or clearings in the YPSC top agar containing S17-1 prey).

### RNA extractions from cells on a surface and RT-PCR

Ten millilitres of *B*. *bacteriovorus* HI strains (grown in PY media) matched to OD_600_ 1.0 were concentrated tenfold by centrifugation at 5,500 x *g* for 10 minutes and resuspended in 1 ml PY. These were incubated for 1 hour at 29°C with shaking at 200 rpm. Two hundred microlitre samples were spread onto 1% agarose Ca/HEPES Petri plates and incubated (either for 4 hours at 29°C or immediately for t = 0), before addition of 5 ml Ca/HEPES 1% phenol 5% ethanol which was agitated, removed and then incubated on ice for at least 45 minutes. Liquid controls of 200 μl were directly added to 5 ml Ca/HEPES 1% phenol 5% ethanol and incubated on ice for at least 45 minutes. Samples were pelleted by centrifugation at 5,500 x *g* for 10 minutes and pellets were stored at -80°C. RNA was isolated from the samples using a Promega SV total RNA isolation kit with the RNA quality being verified by an Agilent Bioanalyser using the RNA Nano kit. Total RNA concentrations were matched to 15 ng/μl for comparison between samples. RT-PCR was performed with the Qiagen One-step RT-PCR kit with the following reaction conditions: One cycle 50°C for 30 minutes, 95°C for 15 minutes, then 25–30 cycles of 94°C for 1 min, 50°C for 1 min, 72°C for 1 min, a 10 minutes extension at 72°C after the 30 cycles, and finally a 4°C hold. Two independent repeats were carried out.

### Quantitative Real-Time RT-PCR

RNA samples extracted as described above were used with Superscript III reverse transcriptase (Invitrogen) to generate cDNA according to the manufacturer’s instructions. Serial dilutions of 1/8 to 1/256 were used as templates for QRT-PCR. PCR products were gel purified on a 2% Agarose gel and extracted with a Sigma gel extraction kit according to the manufacturer’s instructions and 10-fold serial dilutions of this to from 10^−4^ to 10^−9^ were used as template for standard curves. Reactions were carried out with the Fast SYBR green kit (Applied Biosystems) on an Applied Biosystems Quantstudio 6 Flex instrument run by Quantstudio Real Time PCR software v 1.3. Cycling conditions were 1 cycle at 95°C for 20 s followed by 40 cycles of 95°C for 1 s and 60°C for 20 s, then a melt curve of 95°C for 20 s, then 60°C for 60 s and 95°C for 15 s. Primer concentrations were optimized for each template at 30–200 nM and reaction efficiencies were monitored in the software. Only reactions with 90–110% efficiency were used and expression levels were output relative to the standard curve and normalized relative to all reactions per biological repeat.

### Overexpression and purification of dinucleotide cyclase enzymes

Full length WspR D70E and DisA with C-terminal His_6_ tag encoded in pET24a-derived plasmids are from [10]. Full-length DncV with N-terminal His_6_-MBP tag encoded in pET16-derived plasmid is from Kranzusch *et al*. [26]. Full-length Bd0367 i-site mutation with N-terminal His_6_-MBP tag encoded in pET16-derived plasmid is from [10], and the S214D mutant is from this study, constructed by mutagenic PCR. This was achieved using primers 3’ GATTATGTGCTGAGCGAAGTTGG 5’ and 3’ CCCGAATAAGTGGTCGTG 5’ which were phosphorylated with T4 PNK (NEB), used to PCR amplify the pET MBP-Bd0367 R260A, and the PCR product self-ligated using T4 Ligase (NEB).

Canonical GGDEF domains possess a conserved allosteric inhibitory site (I-site) that bind cyclic dinucleotides. We previously showed that, as with many GGDEF enzymes, wild-type GacA from *Geobacter sulfurreducens* co-purifies predominantly with c-di-GMP bound and has low activity when expressed in *E*. *coli*, whereas the R393A I-site mutant of GacA (homologous to R260 in Bd0367) does not purify with bound dinucleotides and exhibits increased *in vitro* activity [10]. We therefore used an I-site mutant (R260A) to assess *in vitro* activity of Bd0367.

Proteins were overexpressed in *E*. *coli* BL21 (DE3) Star cells harboring the pRARE2 plasmid encoding human tRNAs (Novagen). Briefly, an aliquot of the overnight starter culture was re-inoculated into LB with antibiotics (LB/Carb/Chlor for pET16, LB/Kan/Chlor for pET24a) and grown to an OD600 ~0.7, after which cultures were induced with 1 mM IPTG for 10 hr. After centrifugation to isolate the cell pellet, cells were lysed by sonication in lysis buffer (25 mM Tris-HCl (pH 8.2), 500 mM NaCl, 20 mM imidazole, and 5 mM beta-mercaptoethanol). Lysate was then clarified by centrifugation at 10,000 × g for 45 min at 4°C. Clarified lysate was bound to Ni-NTA agarose (Qiagen), and resin was washed with 3 × 20 mL lysis buffer prior to elution with lysis buffer supplemented with 500 mM imidazole. Purified proteins were dialyzed overnight at 4°C into storage buffer (20 mM HEPES-KOH (pH 7.5), 250 mM KCl, 1 mM TCEP, and 5% (v/v) glycerol). Proteins purified in this way were concentrated to ~5–10 mg/mL, flash frozen in liquid nitrogen, and stored at −80°C. Protein purity was assessed by SDS-PAGE (Fig A in S1 Text).

### Extraction of cyclic dinucleotides from *B*. *bacteriovorus* host-independent cells

The method of Bobrov [44] was used to determine the extractable levels of cyclic dinucleotides in axenically grown *Bdellovibrio* cells. Axenically growing HI *Bdellovibrio* strains (mutant and wild type HI) were grown in 50 ml of PY broth from a starting OD_600nm_ 0.2 until they reached a final OD_600nm_ 0.6. Cells were then pelleted by centrifugation and frozen in liquid nitrogen and then processed using Bobrov’s method and 250 μl extraction buffer (40% methanol 40% acetonitrile in 0.1 N formic acid) which was later neutralised with NH_4_HCO_3_. The pellets were dried for transportation and resuspended in 250 μl water and 50 μl of this was used for quantifying.

### LC-MS and Quantification of cyclic dinucleotides from *B*. *bacteriovorus* cell extracts

LC-MS analysis of *B*. *bacteriovorus* cell extracts or cyclic dinucleotide standards was performed using an Agilent 6530 Accurate-Mass Q-TOF LC-MS with an Agilent 1290 Infinity UHPLC. Samples were separated on a Poroshell 120 SB-Aq column (50 mm length x 2.1 mm internal diameter, 2.7 μm particle size, Agilent) at a flow rate of 0.6 ml/min. For analysis of cell extracts, a linear elution program of 0 to 20% B over 4 min with an initial hold at 0% B for the first 0.2 min was used. Solvent A was H_2_O + 0.1% formic acid and solvent B was MeCN. MS data were collected from 0.9 to 2.4 min.

Extract samples were analyzed by MS in the positive ion mode using the range of m/z = 50 to 1100 or 1700. cAG levels were quantified by integrating the m/z = 675.1072 +/- 1 ion extraction over the entire timetable.

### *In vitro* activity assay of dinucleotide cyclases using radiolabeled NTPs

*In vitro* activity assays were performed as previously described [26] as independent technical replicates (n = 3, assays used the same stock enzyme preparation in separate reaction mixtures), with the following modifications. Enzyme (10 μM) was incubated in reaction buffer (50 mM Tris-HCl (pH 7.5), 100 mM NaCl, 10 mM MgCl_2_, and 5 mM dithiothreitol) with 100 μM each NTP substrate and/or nonhydrolyzable analog, and ~0.1 μCi radiolabeled [α-^32^P]-ATP or [α-^32^P]-GTP (Perkin Elmer) at 28°C for 1 hr. After the reaction, the mixture was treated with 20 U of Calf Intestinal Alkaline Phosphatase (NEB) at 28°C for 30 min to digest unincorporated NTPs, followed by heating to 95°C for 30 s to terminate the reaction. The reaction mixture (1 μL) was spotted onto a PEI-cellulose F thin layer chromatography plate (Millipore), and allowed to dry for 15 min at room-temperature. TLC plates were developed using 1 M KH_2_PO_4_ (pH 3.6) as the mobile phase. Plates were dried overnight and radiolabeled products were detected using a phosphor-imager screen (GE Healthcare) and Typhoon Trio+ scanner (GE Healthcare).

## Supporting information

S1 Text Supplementary Material. **Fig A.** SDS-PAGE gel analysis of purified MBP-tagged Bd0367 (MW = 83.9 kDa) and mutant versions. The ladder used was Color Prestained Protein Standard, Broad Range (10–250 kDa, New England Biolabs) and gel was stained with GelCode Blue (Thermo Scientific). **Fig B.** (A) HPLC analysis of nucleotide standards using ~50 μM of each nucleotide. The nucleotides were detected at 254 nm and found to elute in the order of NTPs (1.6 min), 3’,2’-cGAMP (3.2 min), 2’,3’-cGAMP (6.9 min), c-di-GMP (10.0 min), 3’,3’-cGAMP (10.5 min), and c-di-AMP (broad peak from 11–12 min). An unrelated impurity is marked with an *. The HPLC conditions were: flow rate of 0.4 mL/min: 0% B for 5 min, followed by a linear gradient from 0 to 10% B over 1.5 min, hold at 10% B for 2 min, linear gradient from 10 to 30% B over 5 min, followed by a final hold at 100% A for 5 min. Solvent A was 10 mM ammonium acetate + 0.1% acetic acid and solvent B was methanol. (B) HPLC analysis of nucleotides co-purified with Bd0367 WT or I-site (R260A) mutant. Whereas WT enzyme is purified with c-di-GMP and 3’,3’-cGAMP bound, the R260A mutant almost completely eliminates the I-site binding. **Fig C**. HPLC analysis of enzyme reactions with Bd0367 variants shows the switch from primarily cGAMP production for R260A to almost exclusively c-di-GMP production for S214D R260A. A phosphomimic (D74E R260A) shows a slight product shift in favour of c-di-GMP. Two replicate traces are shown for each enzyme reaction, which were conducted using 1:1 ATP/GTP substrates. Nucleotide products were assigned based on comparison to the nucleotide standards (top trace). An unrelated impurity is marked with an *. In some replicates (R260A Rep A), we observed a side product at 13.5 min that could not be assigned. The data table shows manually integrated peak areas that are then normalized to the extinction coefficients for the cyclic dinucleotides to determine the relative product ratios. For c-di-GMP: 23700 M^-1^ cm^-1^, cGAMP: 25050 M^-1^ cm^-1^, c-di-AMP: 27000 M^-1^ cm^-1^ from [45]. The integrated peak area for cGAMP is likely an overestimate, because it includes the cGAMP peak at 10.5 min and a minor c-di-GMP-related peak at 10.4 min that is also present in GTP only enzyme controls, which shows the same mass signals as the main c-di-GMP peak. However, because these two small peaks have overlap and to keep the integration method consistent between enzyme reactions, the cGAMP peak area was determined by integrating over the region containing both peaks. **Fig D.** Epifluorescence phase-contrast microscopy demonstrates that all strains derived from *B*. *bacteriovorus Δbd0367* attach to and enter prey cells; elongation and septation then occurs within prey (bdelloplasts; 4–6 hours). Images are from predatory cultures achieved by mixing the required *B*. *bacteriovorus* HI strain and *E*. *coli* with fluorescently labelled pMal-mCherry to backlight the growing filamentous *Bdellovibrio* cell within. Images are representative of at least two independent experiments. + WT*bd0367* are mutant strains with the wild-type *bd0367* gene restored, replacing the mutated gene. **Fig E.** Plaque assay summary supporting **Fig 5** showing the frequency of different plaque phenotypes observed over 14 independent experiments. Highlighted results show the strains of the *bd0367* deletion (Δ*bd0367*) and the two *bd0367*(S214D) isolates (S214D-1 and -2) consistently did not form plaques (12/13 and 11/13 experiments) apart from the site-directed mutants which infrequently formed a single plaque within the area of HI growth (yellow arrows), predicted to be suppressor strains (although these could not be isolated and occurred only 1/13 assays carried out for S214D-1 and 2/14 assays carried out for S214D-2). Wild-type strains always (14/14 experiments) formed a large area of clearing and reconstructed wild type *bd0367* strains almost always (11/14, 13/14 and 13/13 experiments) demonstrated some areas of clearing or plaques. Representative images of plaque phenotypes are shown to the right and above. **Fig F.** RT-PCR investigating swimming motility gene expression in liquid. RNA was extracted from cultures incubated in liquid for 4 hours and concentrations were matched (see methods). Expression of motility related genes (*crsA*, *fliF* and *fliC5*) and control gene *dnaK* are at a similar level in all strains. Images are representative of two independent repeats.–ve no template negative control, +ve genomic DNA positive control, L- NEB 100bp ladder, sizes shown in bp. **Table A**. Strains and Plasmids. **Table B.** Primers. **Table C.** Values of *n* (number of independently grown HI broth cultures which successfully grew to OD_600_ >0.45) for scoring of cultures for swimming for data for Fig D in S1 Text. **Table D.** Base found in the first position of codon 39 of *fliS* in the plasmid introduced to the wild-type and mutant *bd0367* strains.
(DOCX)

S1 Data cGAMP levels data for Fig 3A. (PZFX)

S2 Data C-di-GMP levels data for Fig 3B. (PZFX)

S3 Data Gliding data for Fig 6. (PZFX)

S4 Data QPCR data for Fig 7. (PZFX)

S5 Data Swimming data for Fig 8. (PZFX)
