# Supplementary material for: Production of 3′,3′-cGAMP by a Bdellovibrio bacteriovorus promiscuous GGDEF enzyme, Bd0367, regulates exit from prey by gliding motility
Source: PLoS Genet. 2022 May 27;18(5):e1010164. doi: 10.1371/journal.pgen.1010164 (PMC9140294; doi:10.1371/journal.pgen.1010164)
Supplement: S1 Text — Fig A. SDS-PAGE gel analysis of purified MBP-tagged Bd0367 (MW = 83.9 kDa) and mutant versions. The ladder used was Color Prestained Protein Standard, Broad Range (10–250 kDa, New England Biolabs) and gel was stained with GelCode Blue (Thermo Scientific). Fig B. (A) HPLC analysis of nucleotide standards using ~50 μM of each nucleotide. The nucleotides were detected at 254 nm and found to elute in the order of NTPs (1.6 min), 3’,2’-cGAMP (3.2 min), 2’,3’-cGAMP (6.9 min), c-di-GMP (10.0 min), 3’,3’-cGAMP (10.5 min), and c-di-AMP (broad peak from 11–12 min). An unrelated impurity is marked with an *. The HPLC conditions were: flow rate of 0.4 mL/min: 0% B for 5 min, followed by a linear gradient from 0 to 10% B over 1.5 min, hold at 10% B for 2 min, linear gradient from 10 to 30% B over 5 min, followed by a final hold at 100% A for 5 min. Solvent A was 10 mM ammonium acetate + 0.1% acetic acid and solvent B was methanol. (B) HPLC analysis of nucleotides co-purified with Bd0367 WT or I-site (R260A) mutant. Whereas WT enzyme is purified with c-di-GMP and 3’,3’-cGAMP bound, the R260A mutant almost completely eliminates the I-site binding. Fig C. HPLC analysis of enzyme reactions with Bd0367 variants shows the switch from primarily cGAMP production for R260A to almost exclusively c-di-GMP production for S214D R260A. A phosphomimic (D74E R260A) shows a slight product shift in favour of c-di-GMP. Two replicate traces are shown for each enzyme reaction, which were conducted using 1:1 ATP/GTP substrates. Nucleotide products were assigned based on comparison to the nucleotide standards (top trace). An unrelated impurity is marked with an *. In some replicates (R260A Rep A), we observed a side product at 13.5 min that could not be assigned. The data table shows manually integrated peak areas that are then normalized to the extinction coefficients for the cyclic dinucleotides to determine the relative product ratios. For c-di-GMP: 23700 M-1 cm-1, cGAMP: 25050 M-1 cm-1, c-di-AMP: [file pgen.1010164.s001.docx]

**Supplementary Material for:**

“**Production of 3′,3′-cGAMP by a *Bdellovibrio bacteriovorus* promiscuous GGDEF enzyme, Bd0367, regulates exit from prey by gliding motility”**

Rebecca C Lowry, Zachary F Hallberg, Rob Till, Tyler J Simons, Ruth Nottingham, Fiona Want, Ming C Hammond*, R Elizabeth Sockett*, Carey Lambert*

| Strain | Genotype/description and use | Reference/  Source |
| --- | --- | --- |
| ***Escherichia coli* strains*:*** |  |  |
| S17-1 | *thi,pro,hsdR*^-^,*hsdM*^+^,*rec*A; integrated plasmid RP4-Tc::Mu-Km::Tn*7*; used as donor for conjugating plasmids into *Bdellovibrio* | (Simon et al., 1983)[6] |
| S17-1::pMal-p2_mCherry | S17-1 strain containing pMal-p2_mCherry plasmid; exports mCherry protein into *E. coli* periplasm, used for backlit microscopy | (Fenton et al., 2010)[1] |
| *Bdellovibrio* strains*:* |  |  |
| *B. bacteriovorus* HID13 | HI isolate of HD100 with a G-A substitution at base 3 in bd0108 | (Lambert et al., 2010)[4] |
| *B. bacteriovorus* HID22 | HI isolate of HD100 with a 42 bp deletion from base 210 to base 252 in bd0108 | (Lambert et al., 2010)[4] |
| *B. bacteriovorus ∆bd0367* | A host-independent *B. bacteriovorus* strain with a silent *bd0367* deletion. | (Hobley et al., 2012)[3] |
| *B. bacteriovorus ∆bd0367* wild type complementation strain | The *B. bacteriovorus* strain *∆bd0367* with *bd0367* inserted back into the genome at the original location using the vector, pK18_*bd0367.* | This study |
| *B. bacteriovorus bd0367(S214D)*-1 | *B. bacteriovorus ∆bd0367* with the *bd0367(S214D)* site-directed mutant gene inserted into the genome using the vector pK18_*bd0367(S214D).* Isolate 1 of the two isolates analysed in this study. |  |
| *B. bacteriovorus bd0367(S214D)*-2 | *B. bacteriovorus ∆bd0367* with *bd0367(S214D)* site-directed mutant gene inserted into the genome using the vector pK18_*bd0367(S214D).* Isolate 2 of the two isolates analysed in this study |  |
| *B. bacteriovorus bd0367(S214D)*-1 wild type complementation strain | *B. bacteriovorus* strain *bd0367(S214D)*-1 with the site-directed mutant gene, *bd0367(S214D)*, fully replaced with the wild type gene, *bd0367*, on the genome. |  |
| *B. bacteriovorus bd0367(S214D)*-1 wild type complementation strain | *B. bacteriovorus* strain *bd0367(S214D)*-1 with the site-directed mutant gene, *bd0367(S214D)*, fully replaced with the wild type gene, *bd0367*, on the genome. |  |
| **Plasmids:** |  |  |
| pK18*mobsacB* | Km^r^ sucrose suicide vector used for conjugation and recombination into *Bdellovibrio* genome | (Schafer et al., 1994)[5] |
| pK18_*bd0367* | pK18*mobsacB* containing *bd0367* and around 1 kb of flanking genomic sequence either side of the gene; for the complementation of *B. bacteriovorus ∆bd0367* and *bd0367(S214D)* strains | This study |
| pK18_*bd0367(S214D)* | pK18*mobsacB* containing *bd0367(S214D)* and around 1 kb of flanking genomic sequence either side of the gene, for creating site-directed mutant *B. bacteriovorus* strain in the previously generated *bd0367* deletion strain (Hobley et al., 2012)[3]. | This study |
|  |  |  |

**Table A**. Strains and Plasmids

| Primer | Sequence | Description |
| --- | --- | --- |
| **Cloning primers** |  |  |
|  |  |  |
| *pK18_bd0367*(F) | **cgttgtaaaacgacggccagtgcca**CGGATTTACCATCGACCAC | Forward primer binding ~1 kb upstream of *bd0367.* To be used with *bd0367(S214D)(R)* to amplify the first half of the *bd0367(S214D)* gene fragment for NEBuilder HiFi DNA Assembly cloning to synthesise pK18_*bd0367(S214D).*  Or to be used with *pK18_bd0367*(R) to amplify wild type *bd0367* with 1 kb flanking DNA regions to generate pK18_*bd0367* via NEBuilder HiFi DNA Assembly cloning.  In **bold** is the overlap complementary to pK18mobsacB for NEBuilder HiFi DNA Assembly. |
| *bd0367(S214D)(R)* | GACAGCACATA**GTC**TCCGAACAAG | Reverse primer binding within *bd0367* allowing mutation of serine 214 to an aspartate residue within the gene product. To be used together with *bd0367*_upstream(F) for NEBuilder HiFi DNA Assembly cloning to generate pK18_*bd0367(S214D).*  In **bold** is the mutant codon which changes the *bd0367* wild type sequence from coding a serine residue at codon 214 to an aspartate residue. |
| *bd0367(S214D)(F)* | CTTGTTCGGA**GAC**TATGTGCTGTC | Forward primer binding within *bd0367* allowing mutation of serine 214 to an aspartate residue within the gene product. To be used together with *bd0367*_downstream(R) for NEBuilder HiFi DNA Assembly cloning to generate pK18_*bd0367(S214D).*  In **bold** is the mutant codon which changes the *bd0367* wild type sequence from coding a serine residue at codon 214 to an aspartate residue. |
| *bd0367*_downstream(R) | **ggaaacagctatgaccatgattacg**AAGTCAGGCTTCTGCCGG | Reverse primer binding ~1 kb udownstream of *bd0367.* To be used with *bd0367(S214D)(F)* to amplify the second half of the *bd0367(S214D)* gene fragment for NEBuilder HiFi DNA Assembly cloning to synthesise pK18_*bd0367(S214D).*  Or to be used with *bd0367*_upstream(F) to amplify wild type *bd0367* with 1 kb flanking DNA regions to generate pK18_*bd0367* via NEBuilder HiFi DNA Assembly cloning.  In **bold** is the overlap complementary to pK18mobsacB for NEBuilder HiFi DNA Assembly. |
| Q5GAAF_F | TACGGCGGGGCCGCCTTCCTGATGGTGTTGACAGAGAC | Forward primer for use with Q5 site-directed mutation kit (NEB) to generate pK18­_*bd0367*(GGAAF) . |
| Q5GGAAF_R | ACGGGCCGGGATGTCGAT | Reverse primer for use with Q5 site-directed mutation kit (NEB) to generate pK18­_*bd0367*(GGAAF) . |
| **RT-PCR primers** |  |  |
|  |  |  |
| *dnaKF* | **TGAGGACGAGATCAAACGTG** | Forward primer binding to *dnaK* open reading frame |

| *dnaKR* | **AAACCAGGTTGTCGAGGTTG** | Reverse primer binding to *dnaK* open reading frame |
| --- | --- | --- |
| *3099F* | **CATCTGCGGGTTTGAGCTAT** | Forward primer binding to *bd3099* open reading frame |
| *3099R* | **CCCACTGCTGACGAAAGATT** | Reverse primer binding to bd*3099* open reading frame |
| *1480F* | **GTAACAGCCAAGGGTCAGGA** | Forward primer binding to *bd1480* open reading frame |
| *1480R* | **GACCTCAGGATGGGCTTTTT** | Reverse primer binding to *bd1480* open reading frame |

**Table B – Primers**


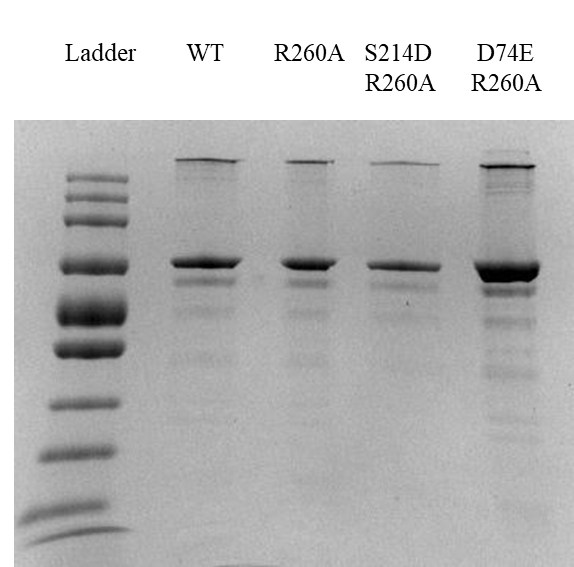


**Fig A.** SDS-PAGE gel analysis of purified MBP-tagged Bd0367 (MW = 83.9 kDa) and mutant versions. The ladder used was Color Prestained Protein Standard, Broad Range (10-250 kDa, New England Biolabs) and gel was stained with GelCode Blue (Thermo Scientific).


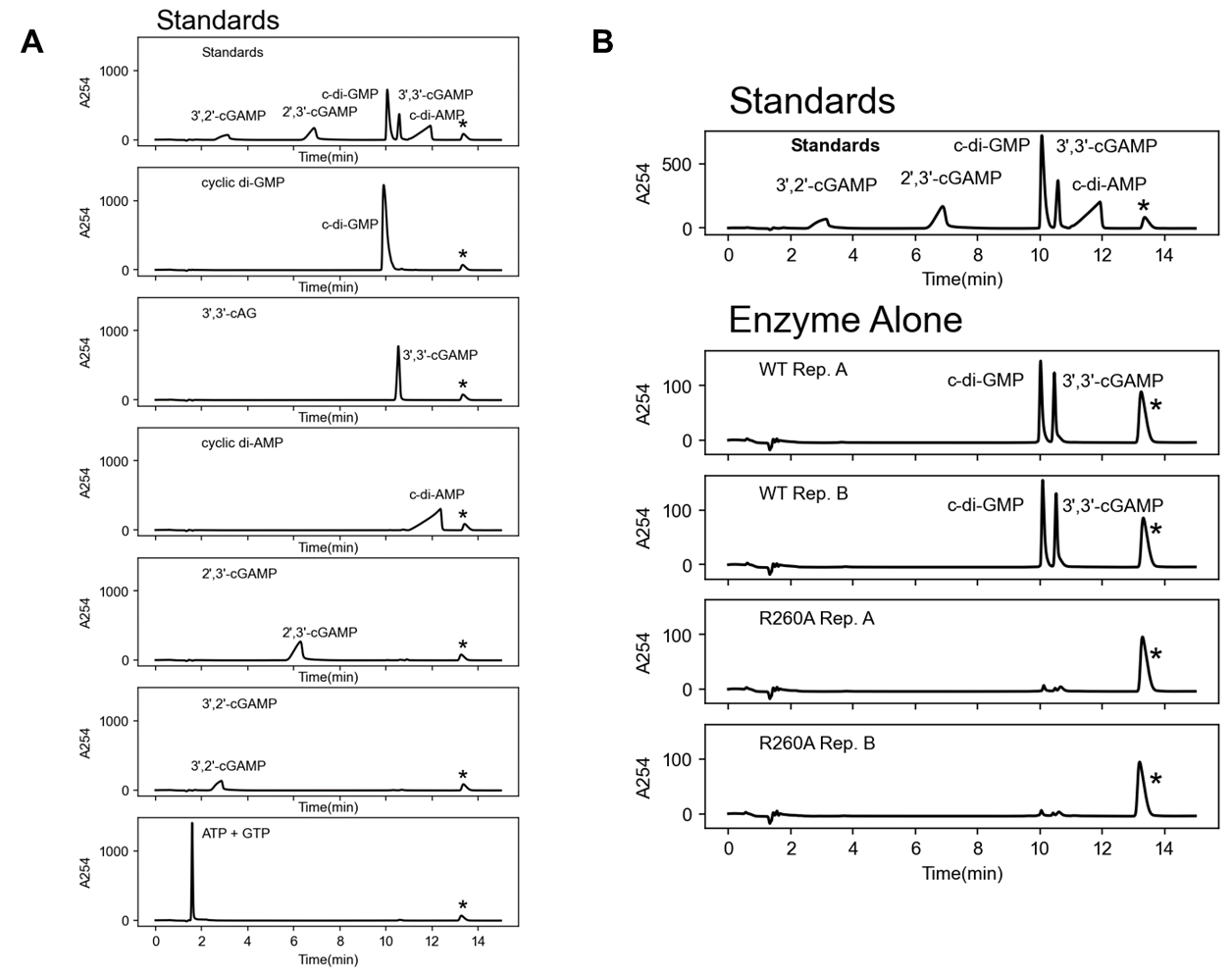


**Fig B.** (A) HPLC analysis of nucleotide standards using ~50 µM of each nucleotide. The nucleotides were detected at 254 nm and found to elute in the order of NTPs (1.6 min), 3’,2’-cGAMP (3.2 min), 2’,3’-cGAMP (6.9 min), c-di-GMP (10.0 min), 3’,3’-cGAMP (10.5 min), and c-di-AMP (broad peak from 11-12 min). An unrelated impurity is marked with an *. The HPLC conditions were: flow rate of 0.4 mL/min: 0% B for 5 min, followed by a linear gradient from 0 to 10% B over 1.5 min, hold at 10% B for 2 min, linear gradient from 10 to 30% B over 5 min, followed by a final hold at 100% A for 5 min. Solvent A was 10 mM ammonium acetate + 0.1% acetic acid and solvent B was methanol

(B) HPLC analysis of nucleotides co-purified with Bd0367 WT or I-site (R260A) mutant. Whereas WT enzyme is purified with c-di-GMP and 3’,3’-cGAMP bound, the R260A mutant almost completely eliminates the I-site binding.

**
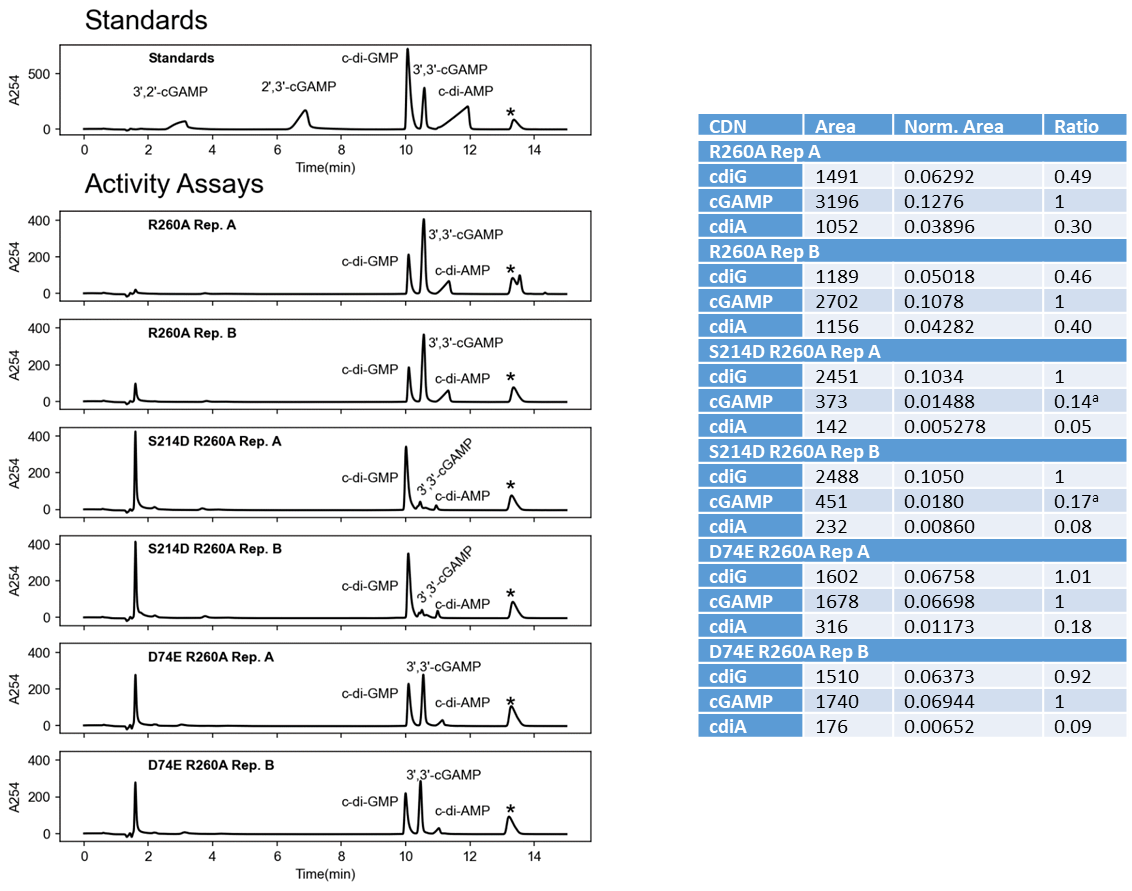
**

**Fig C**. HPLC analysis of enzyme reactions with Bd0367 variants shows the switch from primarily cGAMP production for R260A to almost exclusively c-di-GMP production for S214D R260A. A phosphomimic (D74E R260A) shows a slight product shift in favour of c-di-GMP. Two replicate traces are shown for each enzyme reaction, which were conducted using 1:1 ATP/GTP substrates. Nucleotide products were assigned based on comparison to the nucleotide standards (top trace). An unrelated impurity is marked with an *. In some replicates (R260A Rep A), we observed a side product at 13.5 min that could not be assigned.

The data table shows manually integrated peak areas that are then normalized to the extinction coefficients for the cyclic dinucleotides to determine the relative product ratios. For c-di-GMP: 23700 M^-1^ cm^-1^, cGAMP: 25050 M^-1^ cm^-1^, c-di-AMP: 27000 M^-1^ cm^-1^ from (Gentner et al., 2012) [2]. The integrated peak area for cGAMP is likely an overestimate, because it includes the cGAMP peak at 10.5 min and a minor c-di-GMP-related peak at 10.4 min that is also present in GTP only enzyme controls, which shows the same mass signals as the main c-di-GMP peak. However, because these two small peaks have overlap and to keep the integration method consistent between enzyme reactions, the cGAMP peak area was determined by integrating over the region containing both peaks.


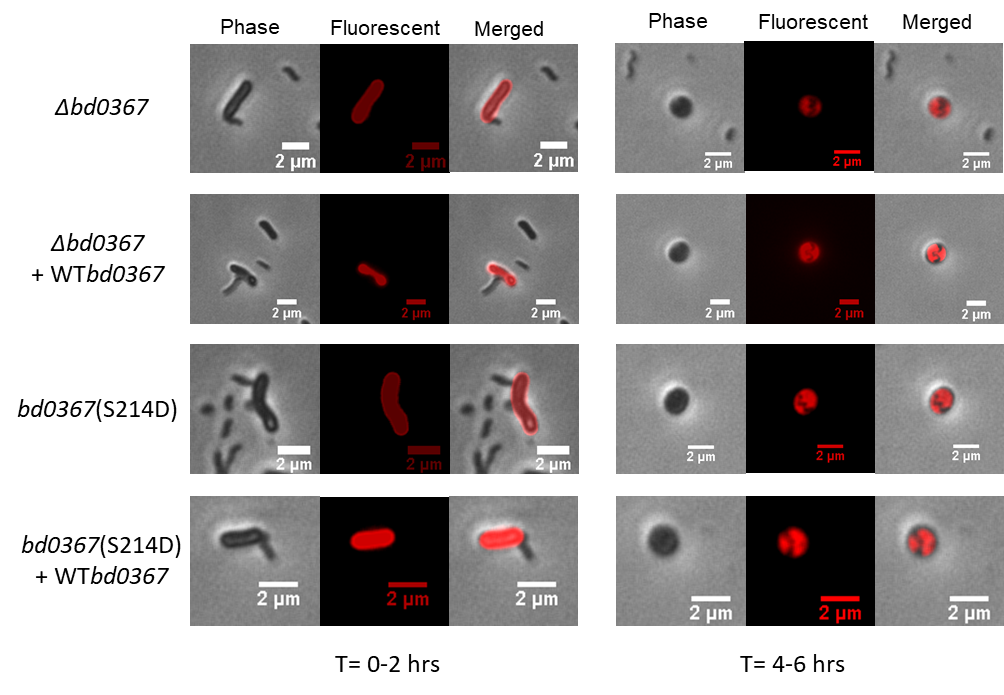


**Fig D.** Epifluorescence phase-contrast microscopy demonstrates that all strains derived from *B. bacteriovorus* *∆bd0367* attach to and enter prey cells; elongation and septation then occurs within prey (bdelloplasts; 4-6 hours). Images are from predatory cultures achieved by mixing the required *B. bacteriovorus* HI strain and *E. coli* with fluorescently labelled pMal-mCherry to backlight the growing filamentous *Bdellovibrio* cell within. Images are representative of at least two independent experiments. + WT*bd0367* are mutant strains with the wild-type *bd0367* gene restored, replacing the mutated gene*.*


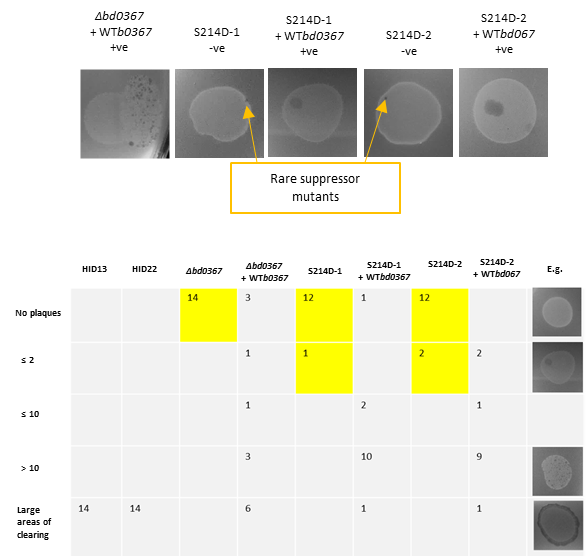


**Fig E.** Plaque assay summary supporting **Fig 5** showing the frequency of different plaque phenotypes observed over 14 independent experiments. Highlighted results show the strains of the *bd0367* deletion (∆*bd0367*) and the two *bd0367*(S214D) isolates (S214D-1 and -2) consistently did not form plaques (12/13 and 11/13 experiments) apart from the site-directed mutants which infrequently formed a single plaque within the area of HI growth (yellow arrows), predicted to be suppressor strains (although these could not be isolated and occurred only 1/13 assays carried out for S214D-1 and 2/14 assays carried out for S214D-2). Wild-type strains always (14/14 experiments) formed a large area of clearing and reconstructed wild type *bd0367* strains almost always (11/14, 13/14 and 13/13 experiments) demonstrated some areas of clearing or plaques. Representative images of plaque phenotypes are shown to the right and above.

| Strain (+*fliDS*) | HID13 | HID22 | *Δbd0367* | *Δbd0367*(+*bd0367*) | *bd0367*S214D | *bd0367*S214D (+*bd0367*) |
| --- | --- | --- | --- | --- | --- | --- |
| Experiment 1 | 11 | 10 | 14 | 8 | 7 | 11 |
| Experiment 2 | 13 | 11 | 7 | 11 | 11 | 4 |
| Experiment 3 | 5 | 7 | 7 | 7 | 7 | 5 |

**Table C.** Values of *n* (number of independently grown HI broth cultures which successfully grew to OD_600_ >0.45) for scoring of cultures for swimming for data for Fig D in S1 Text.


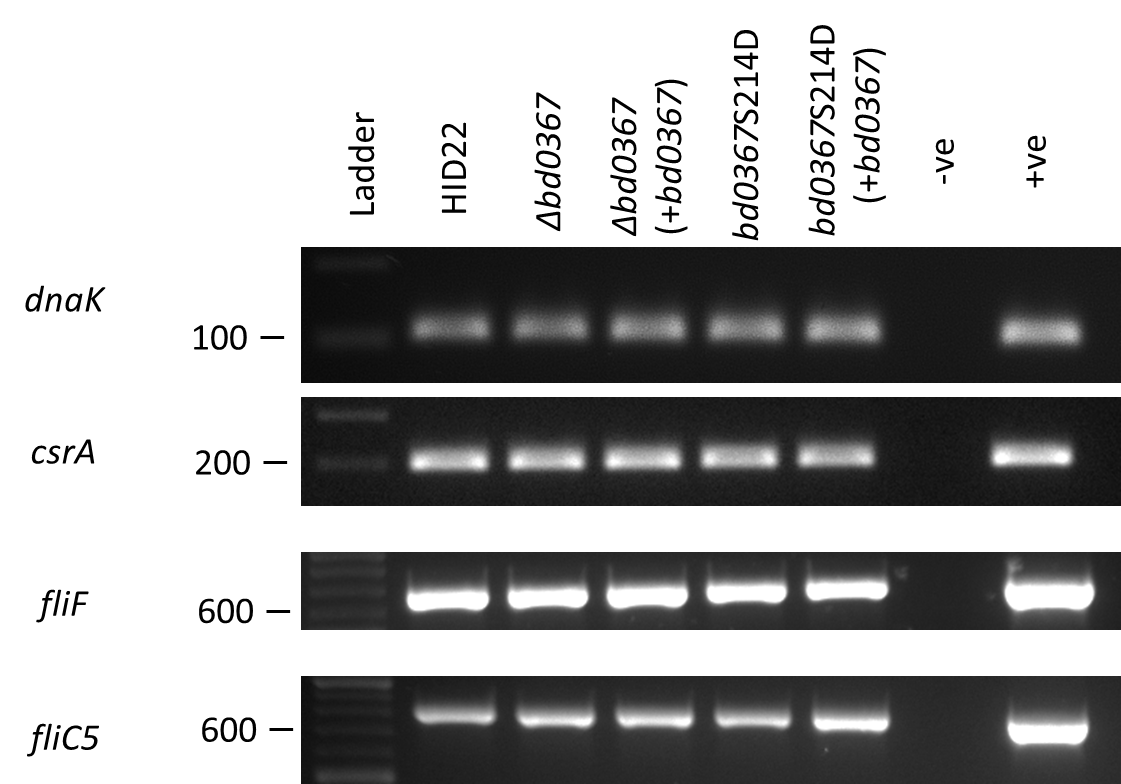


**Fig F.** RT-PCR investigating swimming motility gene expression in liquid. RNA was extracted from cultures incubated in liquid for 4 hours and concentrations were matched (see methods). Expression of motility related genes (*crsA*, *fliF* and *fliC5*) and control gene *dnaK* are at a similar level in all strains. Images are representative of two independent repeats. –ve no template negative control, +ve genomic DNA positive control, L- NEB 100bp ladder, sizes shown in bp.

| Culture | HID13 | HID22 | Δ*bd0367* | Δ*bd0367*(+*bd0367*) | *bd0367*S214D | *bd0367*S214D (+*bd0367*) |
| --- | --- | --- | --- | --- | --- | --- |
| 1 | A | A | T | T | T | A |
| 2 | A | A | T | A | T and A | A |
| 3 | A | A | T | T | T | T |
| 4 | A | A | T | A | T | A |
| 5 | A | A | T | T | T | T |

**Table D.** Base found in the first position of codon 39 of *fliS* in the plasmid introduced to the wild-type and mutant *bd0367* strains.

**Supplementary Discussion.** The wild-type *fliS* codon is AAG encoding a lysine residue and this was often mutated to TAG (a stop codon), upon continued axenic growth of the cultures. For the control strains HID13 and HID22 with a wild type genomic copy of *fliS,* all plasmid copies were wild type. For the Δ*bd0367* strain, all plasmid copies were mutated. The other strains had a mixture of the two. The sequence was determined by PCR amplification of the plasmid sequence (as plasmid extraction from *Bdellovibrio* does not give a high enough yield for sequencing), so the different sequences obtained likely represent a mixed population of wild-type and mutant sequences for these strains. This is demonstrated by repeating the PCRs and sequencing of cultures 1 and 2 for strains Δ*bd0367*(+*bd0367*) and *bd0367*S214D, which gave different results for culture 2 of *bd0367*S214D; the first PCR product had AAG at codon 39, whilst the second PCR product had TAG. The acquisition of mutations in the plasmid in strains capable of tolerating wild-type *fliS* may be due to multiple copies borne on the multi-copy plasmid having a mild deleterious effect. Taken together, the data demonstrate that a wild-type copy of *fliS* cannot be tolerated in a Δ*bd0367* background, and (although being selected against to some extent), can be tolerated in the other genetic backgrounds, including *bd0367*S214D.

**Supplementary Methods**

**Host-independent *B. bacteriovorus* invasion assays**

In order to start *B. bacteriovorus* - *E. coli* infection assays, 1 ml of the desired *B. bacteriovorus* host-independent strain, at an OD_600_ of 1.0 in CaHEPES buffer (originally grown in rich PY media (10 g/L peptone 3 g/L yeast extract) for 16 hours at 29˚C with shaking at 200 rpm, before being pelleted and resuspended in CaHEPES), was mixed with 1.5 ml of fluorescently tagged E. coli S17-1::pMAL-p2_mCherry prey (Fenton et al., 2010) [1] at an OD_600_ of 1.0 in CaHEPES (originally grown in yeast tryptone media containing IPTG at a final concentration of 200 µg/ml). Infections were incubated at 29°C (agitation – 200 rpm) and at time-points: 0, 2, 4 and 6 hours, 10 µl was taken from each infection and cells were immobilised on 1% agarose CaHEPES surface on a microscope slide for analysis on the Nikon Ti-E microscope.

References:

1. FENTON, A. K., KANNA, M., WOODS, R. D., AIZAWA, S. I. & SOCKETT, R. E. 2010. Shadowing the actions of a predator: backlit fluorescent microscopy reveals synchronous nonbinary septation of predatory *Bdellovibrio* inside prey and exit through discrete bdelloplast pores. *Journal of Bacteriology,* 192**,** 6329-35.

2. GENTNER, M., ALLAN, M. G., ZAEHRINGER, F., SCHIRMER, T. & GRZESIEK, S. 2012. Oligomer formation of the bacterial second messenger c-di-GMP: reaction rates and equilibrium constants indicate a monomeric state at physiological concentrations. *J Am Chem Soc,* 134**,** 1019-29.

3. HOBLEY, L., FUNG, R. K., LAMBERT, C., HARRIS, M. A., DABHI, J. M., KING, S. S., BASFORD, S. M., UCHIDA, K., TILL, R., AHMAD, R., AIZAWA, S., GOMELSKY, M. & SOCKETT, R. E. 2012. Discrete cyclic di-GMP-dependent control of bacterial predation versus axenic growth in *Bdellovibrio bacteriovorus*. *PLoS Pathog,* 8**,** e1002493.

4. LAMBERT, C., IVANOV, P. & SOCKETT, L. 2010. A Transcriptional “Scream” Early Response of *E. coli* Prey to Predatory Invasion by *Bdellovibrio* *Current Microbiology* 60**,** 419-427.

5. SCHAFER, A., TAUCH, A., JAGER, W., KALINOWSKI, J., THIERBACH, G. & PUHLER, A. 1994. Small mobilizable multi-purpose cloning vectors derived from the *Escherichia coli* plasmids pK18 and pK19: selection of defined deletions in the chromosome of *Corynebacterium glutamicum*. *Gene,* 145**,** 69-73.

6. SIMON, R., PREIFER, U. & PUHLER, A. 1983. A broad host range mobilisation system for *in vivo* genetic engineering: transposon mutagenesis in gram negative bacteria. *Biotechnology,* 9**,** 184-191.
